# Alginic Acid-Aided Dispersion of Carbon Nanotubes, Graphene, and Boron Nitride Nanomaterials for Microbial Toxicity Testing

**DOI:** 10.3390/nano8020076

**Published:** 2018-01-30

**Authors:** Ying Wang, Monika Mortimer, Chong Hyun Chang, Patricia A. Holden

**Affiliations:** 1 Bren School of Environmental Science and Management, Earth Research Institute, University of California Center for Environmental Implications of Nanotechnology, University of California, Santa Barbara, CA 93106, USA; ywang@bren.ucsb.edu (Y.W.); mmortimer@bren.ucsb.edu (M.M.); 2 University of California Center for Environmental Implications of Nanotechnology, California NanoSystems Institute, University of California, Los Angeles, CA 90095, USA; chonghyun@ucla.edu

**Keywords:** carbon nanotubes, graphene, boron nitride, alginic acid, dispersion, agglomeration, microbiological media, non-covalent functionalization, biocompatibility

## Abstract

Robust evaluation of potential environmental and health risks of carbonaceous and boron nitride nanomaterials (NMs) is imperative. However, significant agglomeration of pristine carbonaceous and boron nitride NMs due to strong van der Waals forces renders them not suitable for direct toxicity testing in aqueous media. Here, the natural polysaccharide alginic acid (AA) was used as a nontoxic, environmentally relevant dispersant with defined composition to disperse seven types of carbonaceous and boron nitride NMs, including multiwall carbon nanotubes, graphene, boron nitride nanotubes, and hexagonal boron nitride flakes, with various physicochemical characteristics. AA’s biocompatibility was confirmed by examining AA effects on viability and growth of two model microorganisms (the protozoan *Tetrahymena thermophila* and the bacterium *Pseudomonas aeruginosa*). Using 400 mg·L^−1^ AA, comparably stable NM (200 mg·L^−1^) stock dispersions were obtained by 30-min probe ultrasonication. AA non-covalently interacted with NM surfaces and improved the dispersibility of NMs in water. The dispersion stability varied with NM morphology and size rather than chemistry. The optimized dispersion protocol established here can facilitate preparing homogeneous NM dispersions for reliable exposures during microbial toxicity testing, contributing to improved reproducibility of toxicity results.

## 1. Introduction

Carbon nanotubes and graphene are carbonaceous nanomaterials (NMs) with exceptional mechanical, electrical, thermal, and optical properties [1,2]. In recent years, boron nitride (BN) NMs have increased in interest due to their many advantages (e.g., higher thermal stability) as compared to carbonaceous NMs [3,4]. With boron and nitrogen atoms substituting for carbon atoms, boron nitride nanotubes (BNNTs) and hexagonal boron nitride flakes (hBNs) can be viewed as analogues of one-dimensional carbon nanotubes and two-dimensional graphene, respectively [3]. Biomedical applications of carbonaceous and BN NMs motivate research regarding their biocompatibility [5,6]. There are also broad concerns for potential human health and environmental risks of NM-enabled products [7,8]. Due to the complexity of NM types, physicochemical characteristics, and toxic mechanisms, high-throughput screening approaches have been proposed to study the comparative toxicity of various NMs to different standard test organisms [9]. Also “low-throughput” studies conducted in more complex environmentally relevant systems are needed for elucidating NM bioeffects at either the community or ecosystem levels [10,11]. In either high- or low-throughput nanoecotoxicology, pro- and eukaryotic microorganisms can serve as excellent model systems for screening and also for assessing mechanisms of NM toxicity.

NM toxicity testing in aqueous environments requires homogeneously dispersing NMs for improved reproducibility of the test results [12,13,14]. In addition to high-throughput mechanistic toxicity testing, homogeneous NM stocks are also used to introduce NMs evenly into more complex media in soil-based studies [15]. However, due to strong van der Waals forces, pristine, unfunctionalized carbonaceous and BN NMs agglomerate which results in heterogeneous exposures and unknown actual exposure concentrations [6,16,17,18,19]. Further, depending on the physiology and ecology of the test organisms and also the NM modes of action, NM agglomeration may either decrease or increase NM bioavailability and biotic effects [20,21,22]. Therefore, NM agglomeration can confound interpretations of observed dose-response relationships and preclude comparing effects across NMs with different physicochemical properties [16].

To disperse NMs for homogenizing exposures, various mechanical methods (e.g., stirring or ultrasonication) and surface functionalization approaches are used [12,13]. Mechanical methods disrupt agglomerates and increase porosity [16], while functionalization incorporates hydrophilic groups to the NM surfaces to improve aqueous dispersibility [6]. Covalent functionalization of NMs (such as introduction of carboxylic groups on carbon nanotube surfaces via oxidation in strong acids [17]), may change the characteristics of NMs (e.g., damaging the π-conjugate system in nanotubes [18]), and thereby may modify the biological effects of NMs [22]. Alternatively, noncovalent modifications, such as using dispersing agents including gum arabic [4], bovine serum albumin [17], Tween-20 [22], sodium dodecylsulfate (SDS) [23], polyvinyl pyrrolidone, Triton X-100 [24], and natural organic matter (NOM) [25,26], have been applied to preserve the intrinsic structural and electronic characteristics of NMs [6,27]. However, many of the common dispersant applications are not environmentally relevant. For example, SDS [23] and Triton X-100 [24] can be toxic and therefore are not suitable for use in toxicity testing of NMs [12], or are applied in unrealistically high concentrations [18,25]. In addition, not all dispersing agents are effective for dispersing multiple types of NMs. NOM, for example, may be widely applicable, but its composition varies significantly, making comparisons across studies difficult [13]. Therefore, to improve the reproducibility and comparability of nanoecotoxicological testing of hydrophobic NMs, there is a need for a nontoxic, and environmentally relevant dispersant with defined composition that would effectively disperse various NMs. Ideally, the dispersant chemistry would reasonably occur in nature, e.g., even within the environment surrounding test organisms as they occur in their natural habitats.

Alginic acid (AA), a natural polysaccharide that is found in the extracellular polymeric substances (EPS) of many bacteria, has been reported to stably disperse multiwall carbon nanotubes (CNTs) by wrapping around the nanotubes while leaving the pristine CNT surfaces partly exposed for bio-interactions [27]. However, the applicability of AA as a dispersant for a range of carbonaceous and BN NMs with different physicochemical characteristics has not been established. AA consists of (1,4)-linked β-d-mannuronic acid and α-l-guluronic acid, and is used in the textile, paper, food, and pharmaceutical industries [28,29]. In addition to being biocompatible [30] and biodegradable [31], AA is environmentally relevant as it is widely distributed in the cell walls of brown algae and secreted by two environmental bacterial genera: *Pseudomonas* and *Azotobacter* [29,32]. Further, fertilizers made from AA have been applied in the field for stimulating soybean growth, indicating likely interactions between soil microflora and AA [33]. Additionally, compared to other natural dispersants, such as NOM [25] and gum arabic [4], AA has a more defined and well characterized chemical composition and structure, which is preferred for a standardized and reproducible protocol [13]. 

Here, we developed a protocol for NM dispersion for microbial toxicity testing. First, we evaluated the effects of AA on two model microorganisms—a prokaryote *Pseudomonas aeruginosa* and a eukaryote, the protozoan *Tetrahymena thermophila*—to confirm the biocompatibility of AA. Then, using AA as the dispersing agent, we optimized the dispersion of one type of CNT as a model hydrophobic NM. The optimized protocol was then used to evaluate AA for aqueous dispersion of three types of CNTs, two types of graphene nanoplatelets (GNPs), BNNTs, and hBN flakes. By using comparable conditions for dispersing NMs with different physicochemical characteristics, we investigated comparative AA-NM interactions and the effects of NM characteristics on NM dispersion stabilities. Additionally, the stabilities of AA-dispersed NMs in two microbiological media were assessed. The protocol for dispersing NMs established here is applicable to uniformly maximizing NM bioavailability during mechanistic studies of microbial nanotoxicity. Given that the protocol appears effective for the several different studied NMs, we believe that our approach could be broadly applicable for studying NM hazards relevant to assessing ecological implications of NMs.

## 2. Results and Discussion

### 2.1. Nanomaterials and Characterization

**Figure 1:**
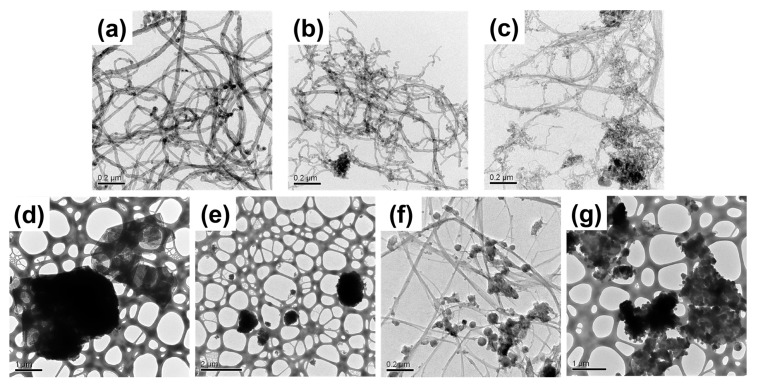
Transmission electron microscopy (TEM) images of pristine multiwall carbon nanotubes (CNT): (**a**) CNT-1; (**b**) CNT-2; and (**c**) CNT-3; graphene nanoplatelets (GNP): (**d**) GNP-1 and (**e**) GNP-2; (**f**) boron nitride nanotubes (BNNT), and (**g**) hexagonal boron nitride flakes (hBN). Scale bars are indicated in each image.

In this study, seven types of NMs were used to test the efficiency of AA in dispersing carbonaceous and BN NMs of varying composition, morphologies, and sizes (Table S1). Pristine NMs were purchased as powders and characterized by transmission electron microscopy (TEM) (Figure 1). The three CNTs were chosen to have different diameters and surface areas as reported by the manufacturer (Table S1), with the outer diameters confirmed by in-house TEM analysis (Figure 1a–c, Table 1) [21,34]. The two GNPs also had significantly different diameters and surface areas (Figure 1d,e, Table 1 and Table S1) [21,34]. The BNNTs had an average measured diameter of 21.9 ± 14.7 nm, comparable to the diameters of CNT-1 and CNT-2 (Figure 1f, Table 1), but considerably larger than the manufacturer reported diameter of 5 nm (Table S1). However, differently from the uniform tube shapes observed in the CNT TEM images, the BNNT TEM images clearly showed the presence of beads, which may be either solidified unreacted boron droplets or byproducts of the BNNT synthesis process (Figure 1f) [35,36]. The pristine two-dimensional hBN flakes had an average measured diameter of 1796 ± 1098 nm (Figure 1g, Table 1), consistent with the manufacturer’s reported size of approximately 1 µm (Table S1).

### 2.2. Biological Effects of Alginic Acid on Microorganisms

AA is an environmentally-relevant dispersant for NMs because it is naturally present in the environment as a constituent of cell walls of brown algae and a component of EPS secreted by certain bacteria, e.g., *Pseudomonas aeruginosa* and *Azotobacter vinelandii* [29,32]. The polymer of AA is comprised of homo-polymeric blocks of either mannuronic or guluronic residues, and hetero-polymeric blocks of alternating guluronic and mannuronic residues [37]. The ratio of these three structural blocks, each with characteristic conformation and functional properties, determines the structure and properties of AA and varies in different organisms. For instance, AA from *Pseudomonas* species is devoid of the poly-guluronic acid sequences, and mannuronic residues are *O*-acetylated to some extent [38]. Due to these structural characteristics and considering the mechanism of AA wrapping onto hydrophobic NMs as discussed below, bacterial AA might not be as efficient a dispersing agent as algal AA. Further, algal AA is readily available due to affordable extraction procedures compared to bacterial AA [39], and thus warrants application as a universal NM dispersant. 

**Figure 2:**
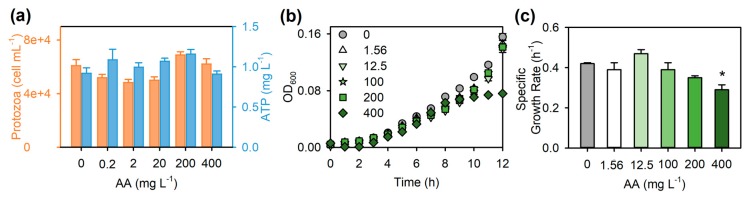
Effects of alginic acid (AA) on the viability of the protozoan *Tetrahymena thermophila* SB210E and the growth of the bacterium *Pseudomonas aeruginosa* PG201. (**a**) Total protozoan cell count (orange bars) and adenosine triphosphate (ATP) content (blue bars) after 1-h incubation with and without AA in Dryl’s medium. There were no significant differences in either the total protozoan cell count or ATP content between the control and the AA treatments (*p* > 0.05); (**b**) Growth curves (measured as optical density at 600 nm, OD_600_, increase with time) and (**c**) specific growth rates of *P. aeruginosa* PG201 (slopes of natural log-transformed OD_600_ values plotted against time, 0–5 h) with and without AA in half-strength 21C medium. Data are mean ± SE (*n* ≥ 4). * *p* < 0.05, as compared to the control.

For AA to be a suitable dispersant of NMs during microbial exposures, it needs to be biocompatible and not induce toxicity in the range of concentrations in nanotoxicity assays. Therefore, effects of AA were assessed on two model microorganisms, a eukaryotic microorganism, the protozoan *Tetrahymena thermophila* SB210E and a prokaryotic microorganism, the Gram-negative soil bacterium *Pseudomonas aeruginosa* PG201. Free AA is not water soluble due to intramolecular hydrogen bonds between the free carboxyl groups of uronic acids. However, ultrasonication increased the water-solubility of AA, likely by breaking the intramolecular H-bonds [37], allowing preparation of well-dispersed and homogeneous AA stock dispersions for toxicity testing. The effects of AA on protozoa were tested in Dryl’s medium [40], which does not support protozoan growth and has a low potential for interfering with NM physicochemical properties and thus is a preferable medium in rapid toxicity screening. AA did not affect the viability of protozoa during a 1-h incubation, as AA did not significantly reduce the total protozoan cell count and adenosine triphosphate (ATP) content compared to the control (Figure 2a). Effects of AA on *P. aeruginosa* growth were assessed in half-strength 21C medium [40], which is suitable for nanotoxicity testing due to its defined composition and low potential for modifying NM physicochemical properties. AA up to a concentration of 200 mg·L^−1^ did not affect bacterial growth compared to the control (Figure 2b,c). However, the specific growth rate for *P. aeruginosa* was significantly lower in the 400 mg·L^−1^ AA treatment than in the control (*p* < 0.05; Figure 2c). This inhibition was unlikely to have been caused by the very slight decrease in pH upon addition of AA to the growth medium (half-strength 21C: pH = 7.2 and 400 mg·L^−1^ AA in half-strength 21C: pH = 7.0). Rather, the higher concentration (400 mg·L^−1^) of AA may have formed complexes with cations in the half-strength 21C medium [27] and thereby decreased the bioavailable nutrient concentrations. AA might have also impeded the diffusion of nutrients from the medium to bacterial cells [41]. Both of these phenomena might have acted simultaneously, causing the slower growth of *P. aeruginosa* in the 400 mg·L^−1^ AA treatment.

In addition to excluding the possible toxicity of the dispersant, it is also important to consider any possible growth stimulatory effects that the added biocompatible dispersant might have on the test organisms. For instance, AA as a polysaccharide might serve as a carbon source to some microorganisms [31,42]. At the highest AA concentration without significant negative effect on bacterial growth (i.e., 200 mg·L^−1^; Figure 2b,c), the carbon content of AA was calculated to be 82 mg·L^−1^. In comparison, the total carbon content of Dryl’s medium—which does not support protozoan growth—is 144 mg·L^−1^ and that of half-strength 21C medium with 3.4 g·L^−1^ glucose is 1379 mg·L^−1^. In the case of Dryl’s medium, AA concentrations that were not growth inhibitory to bacteria would constitute less than 40% of the total carbon in this medium used for *T. thermophila* exposures. Still, AA apparently was not serving as a C source in the exposure time frame, since AA did not affect protozoan concentrations (Figure 2a). It is unlikely that AA contributed to *P. aeruginosa* growth, since less than 6% of the total C in half-strength 21C medium would have arisen from adding AA at any of the non-inhibitory concentrations. This was also confirmed by the *P. aeruginosa* growth assay (Figure 2b,c).

### 2.3. Dispersion Optimization

One type of CNT (i.e., CNT-1) was used to optimize the dispersion protocol by varying the dispersion conditions (including ultrasonication time, AA concentration, and use of AA versus alginate as the dispersing agent). The stability of the obtained AA-dispersed CNT-1 under different conditions was evaluated by monitoring NM agglomeration and sedimentation with time [26,43].

#### 2.3.1. Ultrasonication Time Optimization

**Figure 3:**
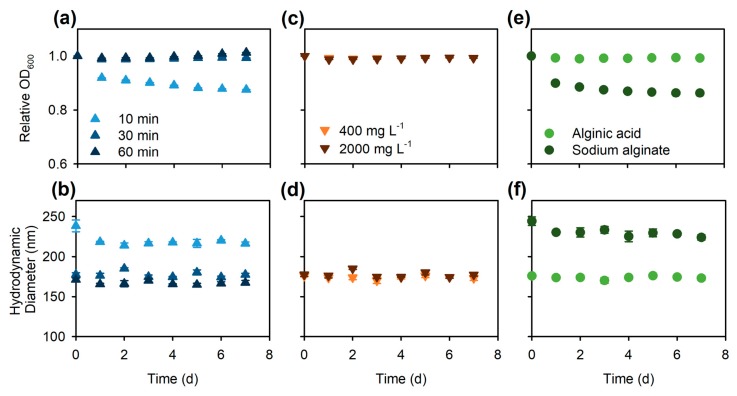
Optimization of nanomaterial dispersion conditions. Multiwall carbon nanotubes (200 mg·L^−1^ CNT-1) were dispersed in Nanopure water under different conditions: (**a**,**b**) using 2000 mg·L^−1^ alginic acid (AA) with varying ultrasonication times (10, 30, and 60 min), or (**c**,**d**) using 30-min ultrasonication with two different AA concentrations (400 and 2000 mg·L^−1^), or (**e**,**f**) using either AA (400 mg·L^−1^) or alginate (derived from 400 mg·L^−1^ AA) with 30-min ultrasonication. The stabilities of the dispersions were evaluated by (**a**,**c**,**e**) the relative optical density at 600 nm (OD_600_) as compared to the OD_600_ at time 0, and (**b**,**d**,**f**) hydrodynamic diameter measured by dynamic light scattering (DLS) over 7 days. Data points are mean ± SE (*n* = 3).

The effect of ultrasonication time (10, 30, or 60 min) on the stability of AA-dispersed CNT-1 was examined for 200 mg·L^−1^ of CNT-1 dispersed with 2000 mg·L^−1^ of AA. This AA concentration of 2000 mg·L^−1^ was used as it is ten times of the CNT-1 concentration which is sufficiently in excess to coat and disperse CNTs [27,28,44]. Thus, at this AA concentration, the CNT dispersion was not limited by AA and ultrasonication time was the single variable. After ultrasonication, the stability of CNT-1 in the sonicated dispersions was followed over time by measuring the optical density at 600 nm (OD_600_; Figure 3a) and the CNT-1 hydrodynamic diameter (Figure 3b). The first measurements, taken within 5 min following ultrasonication, were regarded as the “time 0” results. OD_600_ was used as a proxy for dispersed particle concentration [13]; thus, the relative OD_600_ over time (compared to the OD_600_ at time 0) reflected the dynamic sedimentation process of the CNTs [21]. In the dispersion that was ultrasonicated for 10 min, there was a continuous decrease in OD_600_ over time (up to a ~12% decrease by Day 7; Figure 3a), suggesting significant sedimentation of CNTs following dispersion. By contrast, longer ultrasonication resulted in stable CNT dispersions, indicated by the unchanged OD_600_ over 7 days (Figure 3a). 

Hydrodynamic diameters of CNTs were measured using dynamic light scattering (DLS), which provided information about the particle size distribution and therefore the agglomeration dynamics of CNTs. In DLS, the scattered light fluctuation is related to the particle translational diffusion coefficient, which can be used to estimate a spherical particle hydrodynamic diameter using the Stokes-Einstein equation [13,17]. Therefore, it is worth noting that the measured CNT hydrodynamic diameter is the diameter of a spherical particle that has the same diffusion coefficient as the CNTs [16]. Although it is not the actual size (diameter or length) of the CNTs, the hydrodynamic diameter measured by DLS provides a good estimate for assessing the relative CNT or CNT agglomerate size, and thus is a good indicator for the agglomeration kinetics of CNTs [17,21]. Here, hydrodynamic diameters of CNTs were significantly larger after 10-min ultrasonication with 2000 mg·L^−1^ AA than after 30- and 60-min ultrasonication (both *p* < 0.001), while the hydrodynamic diameters in the two latter dispersions did not differ significantly (*p* = 0.6; Figure 3b). In the dispersion that was ultrasonicated for 10 min, the hydrodynamic diameter of CNTs appeared to decrease by Day 1 compared to the initial (time 0) value (Figure 3b). This was likely caused by the sedimentation of relatively larger agglomerates that were not broken apart by ultrasonication, consistent with the decrease in OD_600_ values (Figure 3a). 

Overall, 30-min ultrasonication was found to be sufficient to efficiently disperse CNT-1 in the presence of 2000 mg·L^−1^ AA. Ultrasonication disrupted CNT bundles and increased porosity by providing a localized shear force [16], and AA sorbed to the surface of CNTs and stabilized smaller agglomerates [17]. Further, ultrasonication contributed by breaking the intramolecular H-bonds in AA [37] and thus facilitating the interactions of AA with CNTs and water which resulted in the stable aqueous dispersion of CNTs.

#### 2.3.2. Alginic Acid Concentration Optimization

To optimize the AA concentration, CNT-1 at 200 mg·L^−1^ was mixed with different concentrations (400 or 2000 mg·L^−1^) of AA, resulting in mass ratios of CNT-1 to AA of 1:2 and 1:10, respectively. After 30-min probe-sonication, there was no sedimentation of CNTs from the dispersions over 7 days as evidenced by no significant decrease in OD_600_ at either AA concentration (Figure 3c). Further, the hydrodynamic diameter of CNT-1 immediately following ultrasonication with 400 mg·L^−1^ AA (176 ± 1 nm) was similar to the diameter of CNT-1 dispersed with 2000 mg·L^−1^ AA (177 ± 2 nm, *p* = 0.8; Figure 3d). In both treatments, CNT hydrodynamic diameters were relatively stable over 7 days, without significant increase, indicating minimal agglomeration of CNTs in the dispersion (Figure 3d). Therefore, the two dispersant concentrations (400 and 2000 mg·L^−1^ of AA) appeared to be equally effective in dispersing CNT-1 with 30-min probe-sonication.

The concentrations of AA reported from studies of bacterial (*P. aeruginosa* and *A. vinelandii*) biofilms ranged from 510 to 6220 mg·L^−1^ [29,32]. Thus, AA concentrations (400 and 2000 mg·L^−1^) tested here for NM dispersion are relevant to actual environmental concentrations. Previously, AA or sodium alginate were used at relatively high concentrations of 2–50 g·L^−1^ for dispersing CNTs and graphene; however, the mass ratios of NMs to AA ranged from 1:0.73 to 1:5 [27,28,44] which were similar to those used in this study. Here, much lower AA concentrations (400 and 2000 mg·L^−1^) were used to produce stable CNT-1 dispersions after ultrasonication (Figure 3c,d). This was achieved by using the low stock CNT concentration of 200 mg·L^−1^ which was sufficient for nanoecotoxicity testing, where the concentration of NMs does not need to exceed 100 mg·L^−1^. In risk assessment, ecological effects of chemicals are characterized using ecotoxicity categories [45,46,47]. Based on these categories, when the L(E)C_50_ value (concentration of chemical that causes lethality or harmful effects in 50% of test organisms) in aquatic toxicity assays is >100 mg·L^−1^, the chemical is classified as “practically nontoxic” or not classified as hazardous. 

Further, in the microbial toxicity assays herein (Figure 2), AA at 200 mg·L^−1^ had no toxic effects on either *T. thermophila* or *P. aeruginosa*, but 400 mg·L^−1^ AA started to significantly inhibit the specific growth rate of *P. aeruginosa*. Taking the toxicity results into consideration, the optimized AA concentration was 400 mg·L^−1^ for dispersion in this study; the NM stock dispersions were thus prepared at 200 mg·L^−1^ with 400 mg·L^−1^ of AA. These concentrations allowed two-fold dilutions of the stocks into 2× concentrated microbiological media, resulting in the highest NM and AA concentrations of 100 and 200 mg·L^−1^, respectively. Therefore, the highest AA concentration used here for microbial toxicity testing (200 mg·L^−1^) is biocompatible. 

#### 2.3.3. Comparison of Alginic Acid and Alginate as Dispersing Agents

Previously, both AA [27] and sodium alginate [28,48] were found to improve the dispersibility of CNTs in aqueous solutions, while sodium alginate was not effective in dispersing graphene [44]. Here, AA (400 mg·L^−1^, pH = 3.4) was converted to sodium alginate (pH = 7.0, [48]) by adding sodium hydroxide, and tested for its efficiency to disperse CNT-1 using 30-min ultrasonication. Compared to AA-dispersed CNT-1, the dispersion prepared using sodium alginate was less stable—with over a 10% decrease in OD_600_ after Day 1—indicating that less than 90% of CNT-1 remained dispersed, followed by a continuous sedimentation afterwards (Figure 3e). The hydrodynamic diameters of sodium alginate-dispersed CNT-1 were also significantly larger than those of the AA-dispersed CNT-1 over 7 days (all *p* < 0.01; Figure 3f). Therefore, AA was more effective than alginate in dispersing CNTs by ultrasonication, possibly due to stronger attachment to the CNT surfaces via hydrophobic interactions [18,27].

### 2.4. Alginic Acid-Aided Dispersion of Carbonaceous and Boron Nitride Nanomaterials by Ultrasonication

**Scheme 1:**
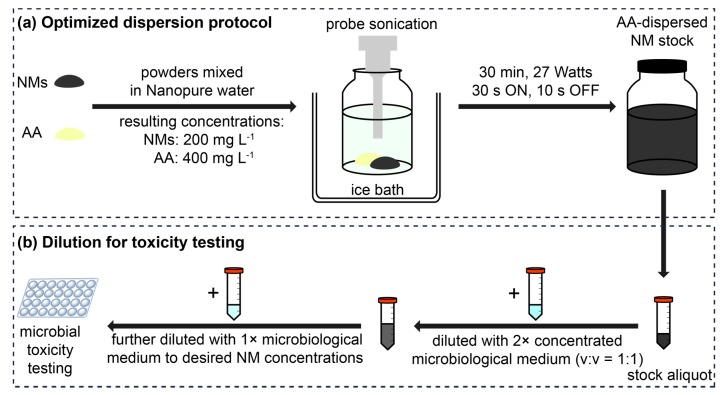
(**a**) Schematic illustration of the optimized protocol for alginic acid (AA)-aided dispersion of carbonaceous and boron nitride nanomaterials (NMs) in Nanopure water by ultrasonication; (**b**) For toxicity testing, the AA-dispersed NM stock in Nanopure water can be diluted into microbiological medium. To avoid dilution of the medium, the AA-dispersed NM stock in Nanopure water is mixed with an equal volume of 2× concentrated medium prior to further diluting in the respective medium.

Based on the results of the dispersion optimization, 30-min probe sonication with 400 mg·L^−1^ AA was deemed optimal for preparing stable CNT-1 (200 mg·L^−1^) dispersions in Nanopure water (Scheme 1a). This optimized dispersion protocol was thus extended to the other six types of NMs, including two other CNTs (CNT-2 and CNT-3), two GNPs (GNP-1 and GNP-2), BNNTs, and hBN, so that the resulting AA-dispersed NM stocks could be diluted into microbiological media for microbial toxicity testing (Scheme 1). All stocks appeared to be homogeneously dispersed after ultrasonication (Figure S1). Zeta (ζ) potentials (Table 1), calculated based on the measured electrophoretic mobility (EPM) values (Table S2) using the Smoluchowski model [12,16], for all seven AA-dispersed NM dispersions in water, were highly negative, in the range of −36.4~−65.3 mV. This indicates that the AA-dispersed NMs had relatively high negative surface charges, which rendered the NMs hydrophilic and provided electrostatic repulsion between NMs to maintain dispersion stability [17]. The homogeneous distribution of NMs in the dispersion was also confirmed by the good linear correlation between the NM concentrations and OD_600_ values (Figure S2). The linear relationship between OD_600_ and NM concentration obeys the Lambert-Beer law, suggesting a high uniformity of the AA-dispersed NMs [25].

**Figure 4:**
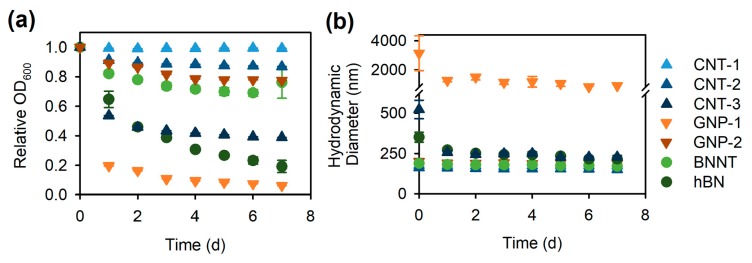
Stability of alginic acid (AA)-dispersed multiwall carbon nanotubes (CNT) CNT-1, CNT-2, and CNT-3, graphene nanoplatelets (GNP) GNP-1 and GNP-2, boron nitride nanotubes (BNNT), and hexagonal boron nitride flakes (hBN) in Nanopure water over 7 days, as evidenced by the time course of (**a**) the relative optical density at 600 nm (OD_600_, as a proxy for dispersed particle concentration) as compared to the OD_600_ at time 0 of the experiment; and (**b**) hydrodynamic diameter as measured by dynamic light scattering (DLS). The dispersion protocol followed the steps for preparing AA-dispersed NM stock in Nanopure water by ultrasonication (Scheme 1a). Data points are mean ± SE (*n* = 3).

Further, the dispersions were characterized for their stability by monitoring NM sedimentation and agglomeration over time. Based on the time course of relative OD_600_ which reflected the sedimentation dynamics, CNT-1 exhibited the highest stability followed by CNT-2, GNP-2, and BNNT (Figure 4a). By contrast, hBN, CNT-3, and GNP-1 were less stable in Nanopure water with less than 40% of NMs remaining dispersed after 7 days (Figure 4a). Similar stability trends were also reflected in the agglomeration kinetics measured by the time course of NM hydrodynamic diameters (Figure 4b). Immediately following ultrasonication, the hydrodynamic diameters of hBN (351 ± 31 nm), CNT-3 (523 ± 57 nm), and GNP-1 (3142 ± 1194 nm) were much larger than those of CNT-1, CNT-2, GNP-2, and BNNT, which were below or close to 200 nm (Figure 4b). Afterwards, the hydrodynamic diameters of CNT-1, CNT-2, GNP-2, and BNNT remained relatively constant over 7 days, indicating no significant agglomeration of dispersed NMs (Figure 4b). By contrast, the hydrodynamic diameters of hBN, CNT-3, and GNP-1 decreased by Day 1 post ultrasonication (Figure 4b). This suggests that some agglomerates in these samples were not easily broken apart by ultrasonication or stabilized by AA and settled out from the dispersion within one day (Figure 4a). Still, smaller agglomerates remained dispersed as hydrodynamic diameters did not increase significantly after Day 1 (Figure 4b).

Among the different types of CNTs, the stability of the dispersions decreased in the order of CNT-1, CNT-2, CNT-3 with CNT-1 being the most stable (Figure 4). Interestingly, this order corresponded to the decreasing outer diameters of CNTs (Table 1): a higher fraction of wider CNTs (with a larger outer diameter), in comparison to thinner CNTs, was dispersed after Day 7 (Figure 4a). Quantitatively, the percentages of AA-dispersed CNTs (indicated by the relative OD_600_) correlated positively with the CNT outer diameters (Figure S3a, *p* < 0.001), and negatively with the CNT specific surface areas (Figure S3b, *p* < 0.001). The lower stability of thinner CNTs might be explained by their larger specific surface area (Table S1) requiring higher concentrations of AA for sufficient surface coverage and stable dispersion of CNTs. Theoretically, based on the specific surface area of CNTs (Table S1), 3333 mg·L^−1^ AA would be needed to cover the surface of 200 mg·L^−1^ CNT-3 at the same level as 200 mg·L^−1^ CNT-1 in 400 mg·L^−1^ AA. As for the two BN NMs, the stability of BNNTs was more similar to those of CNT-1 and CNT-2 which had similar diameters, while hBN flakes displayed similar sedimentation and agglomeration patterns to GNP-1 of similar size (Figure 4, Table 1). When compared based on NM dimensions, the stability of all of the one-dimensional NMs (three CNTs and BNNTs) increased with nanotube outer diameter (Figure S3c, *p* < 0.001), while the stability of the two-dimensional NMs (two GNPs and hBN) decreased with sheet diameter (Figure S3d, *p* < 0.001). Overall, the morphology and size, rather than the chemistry, of carbonaceous and BN NMs, determined their stability in the aqueous dispersions.

### 2.5. Characterization of Alginic Acid Association with Nanomaterial Surfaces

**Figure 5:**
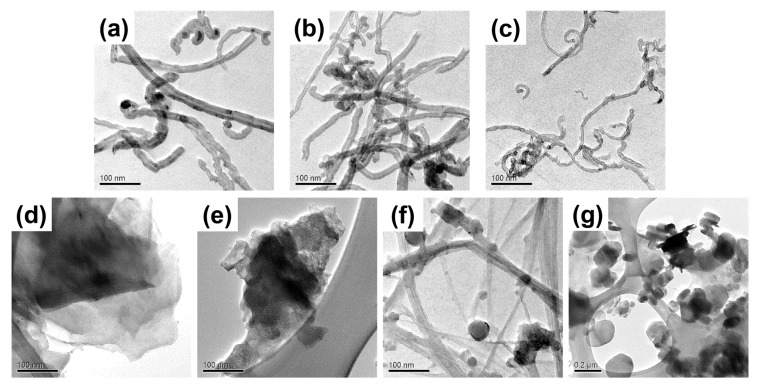
Transmission electron microscopy (TEM) images of alginic acid (AA)-dispersed multiwall carbon nanotubes (CNT) (**a**) CNT-1; (**b**) CNT-2; and (**c**) CNT-3; graphene nanoplatelets (GNP) (**d**) GNP-1 and (**e**) GNP-2; (**f**) boron nitride nanotubes (BNNT), and (**g**) hexagonal boron nitride flakes (hBN). Scale bars are indicated in each image. The dispersion protocol followed the steps for preparing AA-dispersed NM stock in Nanopure water by ultrasonication (Scheme 1a). NM powders for TEM analysis were prepared from the stock dispersions as described in Materials and Methods.

To characterize AA surface associations and NM morphologies after dispersion, the AA-dispersed NMs were filtered, washed, and dried to yield AA-dispersed NM powders. TEM images showed that ultrasonication did not cause significant damage to the structures of NMs (Figure 5). All AA-dispersed one-dimensional NMs (CNT-1, CNT-2, CNT-3, and BNNTs) had similar outer diameters compared to the diameters of the pristine nanotubes (Figure 5 and Figure S4, Table 1). By contrast, two types of the two-dimensional NMs, GNP-1 and hBN, exhibited relatively significant particle size reductions after being sonicated with AA (Figure 5 and Figure S4, Table 1). This was due to the exfoliation of GNP-1 and hBN, which reduced the number of layers and size of the sheets. Thus, the specific surface area of GNP-1 and hBN may have increased after sonication, so that the AA concentration was not high enough for covering the NM surfaces, resulting in less stable GNP-1 and hBN dispersions (Figure 4). However, GNP-2 displayed some larger particles after AA dispersion (Figure S4), possibly due to some extent of agglomeration of the primary particles during preparation of dry powders.

**Figure 6:**
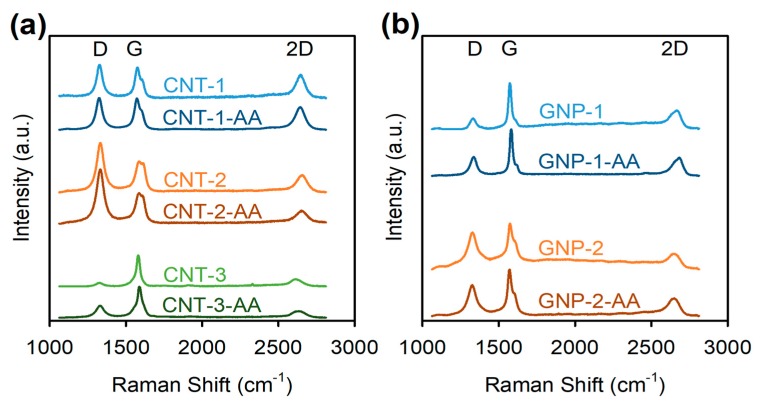
Raman spectra at 633 nm of (**a**) pristine multiwall carbon nanotubes (CNT-1, CNT-2, and CNT-3), alginic acid (AA)-dispersed CNT (CNT-1-AA, CNT-2-AA, and CNT-3-AA); (**b**) pristine graphene nanoplatelets (GNP-1 and GNP-2), and AA-dispersed GNP (GNP-1-AA and GNP-2-AA). Each curve is the average of three replicate spectra taken from different spots in the samples. All spectra were normalized to the G-band intensity. Band positions and band intensity ratios are summarized in Table S3 in the Supplementary Materials. The dispersion protocol followed the steps for preparing AA-dispersed NM stock in Nanopure water by ultrasonication (Scheme 1a). NM powders for Raman analysis were prepared from the stock dispersions as described in Materials and Methods.

AA adsorption to the surface of CNTs and GNPs was examined by Raman spectroscopy. Raman spectra of the pristine CNTs (Figure 6a) showed three prominent bands at around 1328–1332 cm^−1^, 1576–1586 cm^−1^, and 2612–2655 cm^−1^, corresponding to the D, G, and 2D bands, respectively (Table S3). The D band, referred to as the defect band, originates from the breathing modes of sp^2^ carbon rings when proximate to defects or edges [18,49]. The G band is known as the graphite band, attributed to the high-frequency E_2g_ vibrational mode involving sp^2^ carbon atoms in a planar hexagonal lattice [49]. The 2D band is the D band overtone, originating from a two-phonon lattice vibrational process, and is always present but not requiring the presence of defects for its activation [49,50]. After being dispersed by AA, there were no significant changes in the D band position in all three CNTs (Figure 6a, Table S3), indicating there was no covalent binding between AA and CNTs [49], in line with the literature [18,27]. Further, the G (1580 cm^−1^) and 2D (2612 cm^−1^) bands of CNT-3 shifted significantly to higher wavenumbers (1588 and 2633 cm^−1^, both *p* < 0.05; Figure 6a, Table S3) after being dispersed with AA (CNT-3-AA). The up-shifts of the G band and the 2D band correspond to an increase in the energy needed for vibrations to occur, owing to the attachment of AA to the graphite surface of CNTs via hydrophobic interactions and van der Waals attraction forces [49] as reported in previous studies [18,27]. Besides band position shifts, the changes in band intensity ratios are also indicative of the effects of AA surface adsorption on CNT spectroscopic characteristics [50]. Specifically, the ratio of the D and G band intensities (I_D_/I_G_) for AA-dispersed CNT-2 (CNT-2-AA) and CNT-3 (CNT-3-AA) increased significantly compared with that of the pristine CNT-2 and CNT-3, respectively (both *p* < 0.05; Table S3). In addition, significant decreases in the ratio of the 2D and G band intensities (I_2D_/I_G_) for AA-dispersed CNT-1 (CNT-1-AA) and CNT-2 (CNT-2-AA) were also observed by comparing their Raman spectra with those of the pristine CNT-1 and CNT-2 (both *p* < 0.05; Table S3). The increase in I_D_/I_G_ and the decrease in I_2D_/I_G_ both suggest there might be some structural disorder in CNTs caused by the ultrasonication process, or an increased level of long-range ordered crystalline imperfection due to the surface wrapping of AA [18,27,50].

Raman spectra of the pristine GNPs (Figure 6b) confirmed the presence of the characteristic D (1327–1334 cm^−1^), G (1573–1575 cm^−1^), and 2D (2643–2665 cm^−1^) bands specific to GNPs, and are consistent with the results in the literature [44,49]. After being dispersed with AA, the G (1573 cm^−1^) and 2D (2665 cm^−1^) bands of GNP-1 shifted significantly to higher wavenumbers (1582 and 2683 cm^−1^, both *p* < 0.05), and the 2D (2643 cm^−1^) band of GNP-2 also up-shifted by ~7 cm^−1^ (to 2650 cm^−1^, *p* < 0.05), without significant shifts of the D band (Figure 6b, Table S3). In addition, I_D_/I_G_ for GNP-1 increased significantly from 0.23 to 0.40 after the surface modification of AA (both *p* < 0.05; Table S3), which could be ascribed to the increasing fraction of graphene edges or increasing low-edge defects in GNP-1 arising from the ultrasonication process [44,50]. Overall, Raman spectroscopy indicates AA was non-covalently adsorbed to the surfaces of carbonaceous NMs, including both CNTs and GNPs.

**Figure 7:**
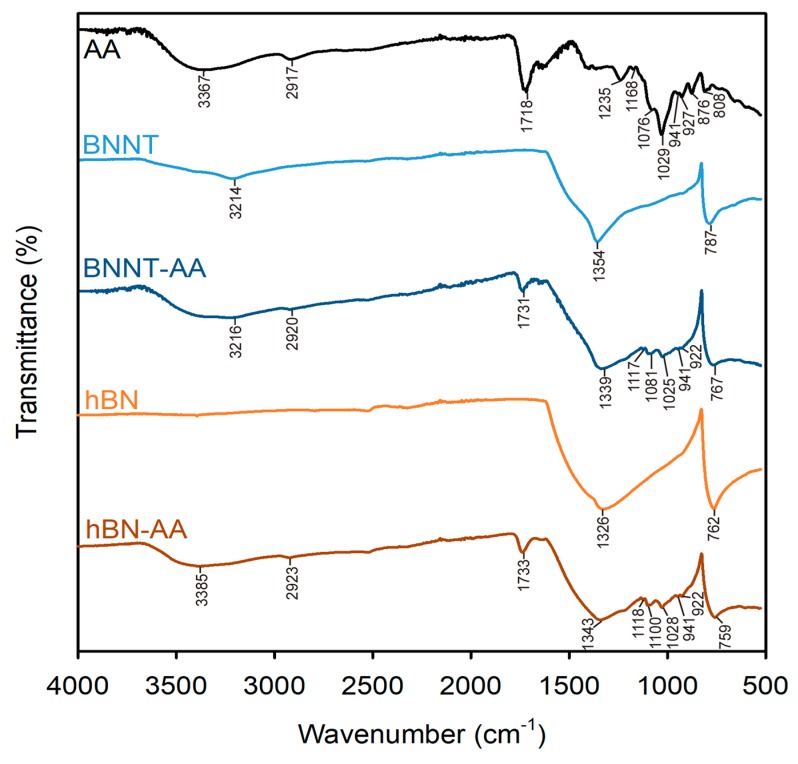
Fourier transform infrared (FT-IR) spectra of alginic acid (AA), pristine boron nitride nanotubes (BNNT), pristine hexagonal boron nitride flakes (hBN), AA-dispersed BNNT (BNNT-AA) and AA-dispersed hBN (hBN-AA). Each curve is the average of three replicate spectra measured from different spots in the samples. Peak assignments are summarized in Table S4 in the Supplementary Materials. The dispersion protocol followed the steps for preparing AA-dispersed NM stock in Nanopure water by ultrasonication (Scheme 1a). NM powders for FT-IR analysis were prepared from the stock dispersions as described in Materials and Methods.

**Scheme 2:**
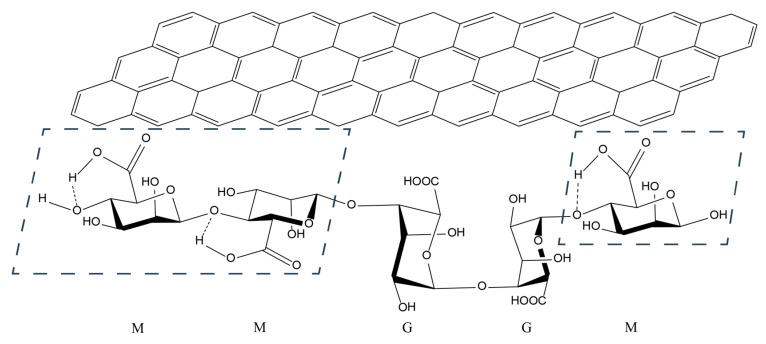
Schematic illustration of hydrophobic interactions between alginic acid (AA; bottom structure) and a graphene sheet (in carbon nanotubes and graphene; top structure). When carbon atoms of the graphene sheet are substituted with boron and nitrogen atoms, it exemplifies a boron nitride sheet (in boron nitride nanotubes and hexagonal boron nitride flakes; not shown here). The molecular structure of AA shows examples of homo-polymeric blocks of mannuronic (M) residues, homo-polymeric blocks of guluronic (G) residues, and hetero-polymeric blocks of alternating M and G residues. The ratio of M and G residues (3:2) shown here indicates the composition of AA used in this study, with ~61% mannuronic acid and ~39% guluronic acid. In M blocks, the intramolecular hydrogen bonding between carboxyl groups and glycosidic residues is favored due to the configuration of C4 and C5 [37], rendering these blocks (indicated by the blue dashed quadrilaterals) relatively hydrophobic and readily interacting with the NM hydrophobic surfaces [27]. Hydroxyl and carboxyl groups of the G blocks are unbound and oriented away from the NM surfaces, making the NM hydrophilic [27].

AA association with hBN and BNNT was characterized by Fourier transform infrared spectroscopy (FT-IR; Figure 7, Table S4). The spectra of pristine hBN flakes and BNNTs had two major peaks characteristic to B–N bond vibrations. The peaks at 1354 and 1326 cm^−1^ in BNNT and hBN spectra, respectively, were assigned to in-plane B–N stretching vibration, while the peaks at 787 and 762 cm^−1^ in BNNT and hBN spectra, respectively, resulted from out-of-plane B–N–B bending vibration [4,51]. The broad peak at 3214 cm^−1^ in the BNNT spectrum could be attributed to hydroxyl groups and indicated surface moisture [51]. The main peaks in the spectrum of AA powder were 3367, 2917, 1718, and 1029 cm^−1^ which were assigned to O–H, C–H, C=O, and C–O groups, respectively [37]. Peaks at 1235, 1168, and 1076 cm^−1^ were attributed to the vibrations in pyranose rings at C–C–H and O–C–H (1235 cm^−1^), C–O–C and C˗OH (1168 cm^−1^), and C–O and C–C (1076 cm^−1^) bonds [52]. In the fingerprint region of carbohydrates (950–750 cm^−1^) four peaks were detected: the weak peaks at 941 and 927 cm^−1^ were assigned to C–O stretching in pyranose rings and α-bond in polysaccharide chains, and the peaks at 876 and 808 cm^−1^ were characteristic of guluronic and mannuronic acid blocks, respectively [37]. The spectrum of AA-dispersed BNNTs (BNNT-AA) indicated a shift of the two major peaks characteristic to B–N bond towards a lower wavenumber, from 1354 to 1339 cm^−1^ and from 787 to 767 cm^−1^. In the spectrum of AA-dispersed hBN (hBN-AA), a shift to higher wavenumber of in-plane B–N stretching vibration (from 1326 to 1343 cm^−1^) was detected and no significant shift appeared in out-of-plane B–N–B bending vibration (762 versus 759 cm^−1^). The shift of the peaks corresponding to pristine BNNTs upon non-covalent functionalization with polysaccharides and peptides has been reported in other studies [4,53] and in this study likely reflects hydrophobic interactions between the mannuronic acid blocks and the surface of BN NMs (Scheme 2). Another indication of the association of AA with BN NMs was the appearance of the peak assigned to hydroxyl groups (3385 cm^−1^) in the hBN-AA spectrum and the broadening of the respective peak (3216 cm^−1^) in the BNNT-AA spectrum, indicating the contribution of AA OH-groups to the FT-IR spectra of AA-dispersed BN NMs. The peak assigned to C=O stretching vibrations in carboxyl groups of AA (1718 cm^−1^) had shifted to a higher wavenumber in the BNNT-AA (1731 cm^−1^) and hBN-AA (1733 cm^−1^) spectra. This shift has been attributed to the cross-linking interaction of –COOH groups [54]. In our study, no covalent or ionic bonding of carboxyl groups was expected; thus, the shift could be explained by intermolecular hydrogen bonding between carboxyl groups and glycosidic bonds in the mannuronic acid blocks (Scheme 2) to facilitate hydrophobic interactions with NM surfaces [37]. Other peaks characteristic of AA were observed in the FT-IR spectra of AA-dispersed BN NMs in the region of 1118–922 cm^−1^ that further confirmed the AA coating on the BN NMs (Figure 7, Table S4).

In summary, Raman and FT-IR spectroscopy indicated that AA was non-covalently adsorbed to NM surfaces via hydrophobic interactions, leaving AA hydroxyl and carboxyl groups oriented away from the NM surface and towards the aqueous medium, contributing to the dispersibility and stability of NMs in water (Scheme 2). TEM analysis confirmed that this non-covalent binding mode helped to preserve the unique structural characteristics of NMs without causing structural damage during the ultrasonication process.

### 2.6. Characterization of Alginic Acid-Dispersed Nanomaterials after Dilution in Microbiological Media

**Figure 8:**
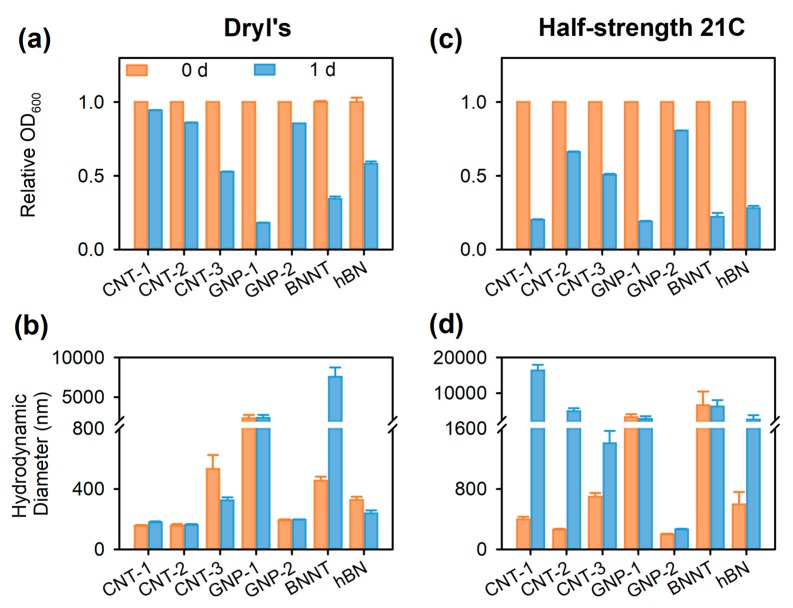
Stability of alginic acid (AA)-dispersed multiwall carbon nanotubes (CNT) CNT-1, CNT-2, and CNT-3, graphene nanoplatelets (GNP) GNP-1 and GNP-2, boron nitride nanotubes (BNNT), and hexagonal boron nitride flakes (hBN) in (**a**,**b**) Dryl’s medium and (**c**,**d**) half-strength 21C medium over one day, as evidenced by the time course of (**a**,**c**) the relative optical density at 600 nm (OD_600_, as a proxy for dispersed particle concentration) as compared to the OD_600_ at time 0 of the experiment, and (**b**,**d**) hydrodynamic diameter as measured by dynamic light scattering (DLS). The dispersion and dilution protocol followed that of Scheme 1. Data bars are mean ± SE (*n* = 3). Note that some error bars (**a**,**c**) do not appear on the graph due to their small values.

The stable AA-dispersed NMs in Nanopure water can be used in microbial toxicity assays directly without further separation of unbound AA, due to the biocompatible and non-toxic nature of AA in the concentration range tested, as described above. For toxicity testing, the stable NM stocks prepared in Nanopure water (Scheme 1a), can be diluted into microbiological media to the desired testing concentrations (Scheme 1b). Since the diluted NMs in microbiological media would be inoculated with microorganisms immediately, and knowing that microbial cells and their secreted extracellular compounds would be expected to change the dispersion stability of NMs, achieving the long-term stability of NMs in microbiological media was not the aim. Still, NM dilutions in two representative microbiological media (i.e., Dryl’s medium and the half-strength 21C medium) were characterized to gain a better understanding of how the physicochemical properties of the NMs influenced their dispersion stabilities in media with different ionic strengths. According to the relative OD_600_, CNT-1 was the most stable NM in Dryl’s medium (ionic strength 0.022 M) with ~95% of CNT-1 remaining dispersed after Day 1, followed by CNT-2, GNP-2, hBN, CNT-3, BNNT, and then GNP-1 (Figure 8a). This trend followed the stability sequence of AA-dispersed NMs in water, except that fewer BNNTs (~35%) were dispersed in Dryl’s medium than in water (~83%) after one day (*p* < 0.001; Figure 4a and Figure 8a). The reduced stability of BNNTs in Dryl’s medium was also reflected in their significantly larger mean hydrodynamic diameter (457 ± 24 nm) than that in water (189 ± 3 nm; *p* < 0.05; Figure 4b and Figure 8b). Continuous agglomeration of BNNTs in Dryl’s medium was reflected by an increase in hydrodynamic diameter to larger than 7500 nm after one day post dilution, in contrast to their stable dispersions in water (*p* < 0.05; Figure 4b and Figure 8b). However, for the other six AA-dispersed NMs, there were no significant differences in their hydrodynamic diameters in Dryl’s medium and in water, either immediately following dilution or on Day 1 (*p* > 0.05; Figure 4b and Figure 8b).

In half-strength 21C medium (ionic strength 0.042 M), significant sedimentation and agglomeration of AA-dispersed NMs were observed relative to water and Dryl’s medium (Figure 8c,d). Specifically, significantly lower amounts of NMs remained in dispersion after one day upon dilution into half-strength 21C medium compared to those in water or Dryl’s medium (all *p* < 0.05; Figure 4a and Figure 8a,c). Agglomeration of CNT-1 and CNT-2 occurred immediately after dilution into half-strength 21C medium, as evidenced by significantly larger hydrodynamic diameters (401 ± 31 and 264 ± 8 nm, respectively) compared to those in water (176 ± 1 and 162 ± 3 nm, respectively; both *p* < 0.05; Figure 4b and Figure 8d). The hydrodynamic diameters of CNT-1, CNT-2, CNT-3, and GNP-2 all increased significantly in one day after dilution in half-strength 21C medium (all *p* < 0.05) and were larger than the diameters in water or Dryl’s medium (all *p* < 0.001; Figure 4b and Figure 8b,d). However, among all AA-dispersed NMs, GNP-2 exhibited the highest level of stability in half-strength 21C medium, with ~81% GNP-1 remaining dispersed with the smallest hydrodynamic diameter (269 ± 5 nm) after one day (Figure 8c,d).

Another significant trend was the decrease in the ζ potential for all seven AA-dispersed NMs after being diluted into Dryl’s and half-strength 21C media compared with their ζ potentials in water (all *p* < 0.01), except for AA-dispersed CNT-3 in half-strength 21C medium (*p* = 0.08; Table 1). This decline, reflecting decreasing surface charges, could be attributed to charge shielding by cations in microbiological media [12] or the possible desorption of surface-bound AA from NMs [27]. It has been found that the coupling between AA and metal cations in the aqueous solution could hinder the wrapping of AA around CNTs [27]. Still, the ζ potentials of all AA-dispersed NMs in both Dryl’s and half-strength 21C media (Table 1) were either more negative than (*p* < 0.05) or close to (without significant difference, *p* > 0.05) the ζ potential of AA (−29.3 ± 0.5 mV), and none was less negative than −30 mV (*p* > 0.05) which is considered as the cutoff ζ potential value of a stable dispersion [16].

Further, the ζ potentials of AA-dispersed NMs in Dryl’s and half-strength 21C media did not differ significantly (*p* > 0.05), except for AA-dispersed GNP-2 which had a more negative ζ potential in half-strength 21C medium than in Dryl’s medium (*p* < 0.001; Table 1). However, based on the results of sedimentation and agglomeration dynamics (Figure 8), more pronounced agglomeration of AA-dispersed NMs was observed in half-strength 21C medium than in Dryl’s medium. The tendency of NMs to agglomerate depends on the balances between two counteracting forces, electrostatic repulsion and van der Waals attraction, according to the DLVO theory [12,13,16,17,19,21]. The repulsive forces depend on the electrical double layer, which has two important features, ζ potential (reflecting surface charge) and the thickness of the electrical double layer [16]. A decrease in either the absolute value of ζ potential or the electrical double layer thickness will lead to a decline in repulsive forces [16]. Once repulsive forces diminish relative to the attractive van der Waals forces, NMs will agglomerate. Here, despite the similar ζ potentials of AA-dispersed NMs in both microbiological media, compared to Dryl’s medium, half-strength 21C medium had a significantly higher ionic strength, which could compress the NM electrical double layer and thus shrink the repulsive forces between NMs. As the repulsive forces were overwhelmed by the attractive van der Waals forces, the van der Waals attraction became the dominant force binding NMs together, thus leading to the more significant NM agglomeration observed in half-strength 21C medium.

Additionally, the cations in microbiological media might have resulted in dissociation of AA from NM surfaces due to AA complexation with cations [27]. This could have further reduced the steric repulsion between AA-dispersed NMs and decreased the dispersion stability [12]. To demonstrate the role of AA in ensuring the stability of NM dispersions, the stability of CNT-1 dispersed with either 100 or 2000 mg·L^−1^ AA in microbiological media was measured (Figure S5). The decrease in AA concentration (from 400 to 100 mg·L^−1^) decreased the stability of the resulting AA-dispersed CNT-1 in microbiological media. For example, in Dryl’s medium, the hydrodynamic diameter of CNT-1 dispersed with 100 mg·L^−1^ AA (284 ± 6 nm) was significantly larger than that of CNT-1 dispersed with 400 mg·L^−1^ AA (182 ± 4 nm, *p* < 0.001). However, minimal sedimentation (less than 4%) and agglomeration of CNT-1 dispersed with 2000 mg·L^−1^ AA were observed after dilution into half-strength 21C medium, with the hydrodynamic diameter slightly increasing from 181 ± 1 nm to 247 ± 9 nm after one day. The increase in AA concentration was effective in reducing NM agglomeration in microbiological media, particularly in half-strength 21C medium, possibly due to two reasons: (i) there was more AA adsorbed to the NM surface, resulting in higher electrostatic and steric repulsive forces; (ii) there was more free AA available to associate with cations in the medium which otherwise would have shed the NM surface-bound AA causing NM destabilization. These results suggested that higher AA concentrations would have increased the dispersion stability of the tested NMs in microbiological media. However, considering that the dispersions were prepared for microbial toxicity testing, we did not use higher AA concentrations than that which was proven to be biocompatible and which provided sufficiently stable NM dispersions in Nanopure water used as stocks (Section 2.3.2).

## 3. Materials and Methods

### 3.1. Nanomaterials and Characterization

Three types of CNTs (CNT-1, CNT-2, and CNT-3) and two types of GNPs (GNP-1 and GNP-2) were purchased from Cheap Tubes Inc. (Grafton, VT, USA). BNNTs and hBN flakes were purchased from Sigma-Aldrich (St. Louis, MO, USA). All NM powders were used as received without further purification (Table S1). NMs were characterized by transmission electron microscopy (TEM; JEOL 1200 EX, Peabody, MA, USA) for material morphology and primary size. For that, samples were dispersed in deionized (DI) water at a concentration of 1 g·L^−1^ by bath sonication (Branson 2510, Danbury, CT, USA) at 40 kHz for 15 min, diluted to 50 mg·L^−1^ in DI water and further sonicated for 15 min. Then a drop of the diluted suspension was placed on a TEM grid and imaged by TEM after drying at room temperature. All diameters of pristine and AA-dispersed NMs were determined by analyzing TEM images using ImageJ (NIH) software. The average diameters of NMs were derived from measuring over 100 particles of each NM within a representative area.

### 3.2. Preparation of Stock Dispersions

Alginic acid (AA, ~61% mannuronic acid and ~39% guluronic acid, molecular weight ~240 kD) from the brown alga *Macrocystis pyrifera* was purchased from Sigma-Aldrich (St. Louis, MO, USA). An AA stock (1 g·L^−1^) was prepared by weighing AA powder into a sterile glass jar (118 mL) and adding sterile Nanopure water (resistivity > 18.2 MΩ·cm, Thermo Scientific Barnstead, Waltham, MA, USA). The jar contents were probe-sonicated for 30 min in an ice bath using a Cole-Parmer 750-W Ultrasonic Homogenizer (Vernon Hills, IL, USA) with a 13 mm diameter probe and replaceable tip at 40% amplitude powder (~27 Watts), with pulsing for 30 s on and 10 s off. Release of titanium (Ti) particles from the tip of the probe was noted after sonication, which was unavoidable. Titanium particles were considered inert and found to settle at the bottom of the jar. The transfer of Ti particles from the sonicated stock dispersions was avoided by not disturbing the bottom layers of the stock dispersions and pipetting from the stably dispersed upper fraction when preparing dilutions as described below. The AA stock in Nanopure water was diluted in protozoan medium (Dryl’s) and bacterial growth medium (half-strength 21C) for toxicity assessment. To avoid dilution of the medium, the AA stock solution in Nanopure water was mixed with an equal volume of 2× concentrated medium prior to further diluting in the respective medium.

For NM stock preparation, the NM and AA powders were transferred into a sterile glass jar in a glove box, to minimize occupational hazards associated with handling of NM powders, and weighed on an analytical balance in a fume hood. Sterile Nanopure water was added to the powders to achieve a resulting NM concentration of 200 mg·L^−1^. The dispersions were probe-sonicated in an ice bath as described above. In the case of CNT-1, sonication durations (10, 30, and 60 min) and AA concentrations (400 and 2000 mg·L^−1^) were varied to optimize the dispersion protocol. To compare AA and alginate for their effectiveness in dispersing NMs, two AA suspensions (both at 400 mg·L^−1^) were prepared by weighing AA powder into sterile Nanopure water in two glass jars. An appropriate amount of sodium hydroxide solution (1 mol·L^−1^) was added to one of the AA suspensions until the pH changed from 3.4 to 7.0, indicating the formation of sodium alginate [48]. Then CNT-1 powder was added into the jars containing AA and sodium alginate suspensions, both at a final CNT-1 concentration of 200 mg·L^−1^. Both samples were probe-sonicated for 30 min at the same settings as above. For other NM stock dispersions, 400 mg·L^−1^ AA was used and the sonication time was 30 min.

### 3.3. Effects of Alginic Acid on Microorganisms

The effects of AA on the protozoan *Tetrahymena thermophila* were evaluated by exposing *T. thermophila* SB210E, pre-grown in SSP medium as described previously [40], to AA (0, 0.2, 2, 20, 200, and 400 mg·L^−1^) in Dryl’s medium (1 mmol·L^−1^ NaH_2_PO_4_, 1 mmol·L^−1^ Na_2_HPO_4_, 2 mmol·L^−1^ sodium citrate, and 1.5 mmol·L^−1^ CaCl_2_, pH 7.0, [40]), at 30 °C in a humidity chamber. After 1 h exposure, samples were taken and fixed with 2.5% glutaraldehyde for cell counting in a hemocytometer. Cell viability was determined by measuring the ATP content, as described before [55]. Briefly, ATP was extracted from protozoan samples with an equal volume of ice cold 4% trichloroacetic acid (TCA) in 4 mmol·L^−1^ ethylenediaminetetraacetic acid (EDTA) disodium salt dihydrate and samples were stored at −20 °C until analysis. For ATP analysis, 100 μL of Tris–EDTA buffer (0.1 mmol·L^−1^ Tris, 2 mmol·L^−1^ EDTA, adjusted to pH 7.75 with acetic acid) was pipetted into the wells of a white 96-well microplate and 5 μL of each thawed sample was added into two wells containing the buffer. Then 100 μL of ATP Assay Mix (Sigma-Aldrich, St. Louis, MO, USA), diluted 100× with ATP Assay Mix Dilution Buffer (Sigma-Aldrich, St. Louis, MO, USA), was added to the diluted samples in the microplate wells. Luminescence emission (*RLU*_sample_) was measured immediately using a Cytation 3 microplate reader (Biotek Instruments, Winooski, VT, USA). 10 μL of ATP (2 × 10^−6^ mol·L^−1^) was then added as an internal standard and luminescence was recorded again (*RLU*_ATP standard_). The concentration of ATP in each sample (*C*_ATP_, in mg·L^−1^) was calculated according to the following equation:

(1) $$C_{\mathrm{ATP}}=\frac{{RLU}_{\mathrm{sample}}}{{RLU}_{\mathrm{ATP}\;\mathrm{standard}}}{\times C}_{\mathrm{ATP}\;\mathrm{standard}}$$

where *C*_ATP standard_ is the concentration of ATP standard in the reaction mixture, in mg·L^−1^. Two independent biological replicates were conducted, each with eight and two replicates for cell counting and ATP measurement, respectively.

The effects of AA on the Gram-negative bacterium *Pseudomonas aeruginosa* PG201 were investigated by monitoring bacterial growth with and without AA (0, 1.56, 12.5, 100, 200, and 400 mg·L^−1^) in half-strength 21C medium [40] (0.5 g·L^−1^ NH_4_Cl, 1.725 g·L^−1^ Na_2_HPO_4_·7H_2_O, 1.38 g·L^−1^ KH_2_PO_4_, 1% *v*/*v* Hutner’s mineral base [45], and 3.4 g·L^−1^ filter-sterilized glucose as a carbon source, pH 7.2). Exponential-phase *P. aeruginosa,* grown as described previously [40,56], was used to inoculate 200 μL of half-strength 21C medium with or without AA in the wells of a 96-well microplate. The 96-well microplate was incubated at 30 °C, 200 rpm in a Synergy 2 Multi-Mode microplate reader (Biotek Instruments, Winooski, VT, USA) and optical density at 600 nm (OD_600_) was measured regularly over time. Four independent biological replicates were conducted.

### 3.4. Nanomaterial Dispersion Stability Characterization

Agglomeration kinetics were studied by measuring the change of NM hydrodynamic diameter over time using disposable cuvettes (DTS0012) via DLS using a Zetasizer Nano-ZS90 (Malvern Instruments Ltd., Malvern, UK), with a 633 nm He-Ne laser at a 90° scattering angle. The laser power was automatically attenuated by the system. Measurements were performed at 20 °C. The parameters for dispersant were approximated as those of water (viscosity: 1.0031 cP; refractive index: 1.330; dielectric constant: 80.4). The NM hydrodynamic diameters were determined as the intensity-weighted mean diameters (i.e., z-average diameters) by cumulants analysis using Malvern Zetasizer software version 7.11 (Malvern Instruments Ltd., Malvern, UK). The dynamic sedimentation process of NMs in the dispersion was monitored by measuring OD_600_ (as a proxy for dispersed particle concentration) using a UV-1800 spectrophotometer (Shimadzu, Kyoto, Japan). DLS and OD_600_ measurements were made daily from 0 to 7 days, and three replicate samples were analyzed.

The agglomeration and sedimentation patterns of AA-dispersed NMs were also tested in two representative microbiological media (i.e., Dryl’s medium and half-strength 21C medium). Specifically, sterile media were filtered through 0.22 μm membrane filters (Millipore, Billerica, MA, USA) to remove any contaminants and dust. Aliquots of the AA-dispersed NM stocks were then diluted into either Dryl’s medium or half-strength 21C medium for DLS and OD_600_ measurements. The OD_600_ of both Dryl’s and half-strength 21C media alone was monitored over time as well to confirm there was neither contamination nor interfering OD_600_ from media [13]. The EPM values of AA-dispersed NMs in water and two media were measured using folded capillary cells (DTS1070) by the Zetasizer Nano-ZS90 (Malvern Instruments Ltd., Malvern, UK). The ζ potentials were calculated based on the EPM values using the Smoluchowski model [16] by Malvern Zetasizer software version 7.11. Measurements were performed for three replicate samples.

### 3.5. Characterization of Alginic Acid Association with Nanomaterials

AA-dispersed NMs were collected on 0.2 μm alumina membranes (Whatman Anodisc) followed by washing with Nanopure water three times. Since the mean hydrodynamic diameters of NMs were determined to be around 200 nm or larger (Figure 4b), no significant NM loss through the membranes was expected. The filtrates contained no significant amounts of NMs as confirmed by OD_600_ measurements, since the OD_600_ value was shown to have a positive linear relationship with NM concentration (Figure S2). The AA-dispersed NM powders were scraped from the membranes, transferred into sterile glass vials, dried overnight at room temperature, and characterized by TEM (JEOL 1200 EX, Peabody, MA, USA) for material morphology and primary size. Raman and FT-IR spectroscopy were used to determine the adherence of AA to the NM surfaces. The powders of pristine CNTs, GNPs, and AA-dispersed CNTs and GNPs were placed on microscope glass slides and analyzed using a LabRAM ARAMIS Raman confocal microscope (HORIBA Jobin Yvon Inc., Edison, NJ, USA). The Raman spectra were acquired from 1100 to 2800 cm^−1^ at room temperature with a He-Ne 633 nm laser as the excitation source. Three replicate spectra were acquired from different spots on each sample. FT-IR spectra were recorded using a Nicolet iS10 FT-IR spectrometer (Thermo Fisher Scientific, Waltham, MA, USA) equipped with a Smart Diamond ATR accessory. The powders of AA, pristine BNNTs, hBN, and AA-dispersed BNNTs and hBN were measured in three different spots in the range of 4000–500 cm^−1^.

### 3.6. Statistical Analyses

Data are shown as the mean ± SE (standard error). Homogeneity of variance of the data was examined using Levene’s test. Means were compared using a *t*-test assuming a two-tailed distribution for one sample or two independent samples, or one-way analysis of variance (ANOVA) with Tukey’s or Games-Howell post hoc multiple comparisons for more than two samples. A statistically significant difference was assumed at *p* < 0.05. The bacterial specific growth rate was calculated as the slope of a regression line from the linear region of the plot of the natural logarithm of OD_600_ versus time. Raman spectral data were processed using PeakFit v4.12 software (Systat Software Inc., San Jose, CA, USA) and FT-IR spectral data were processed by Thermo Scientific OMNIC Series Software (Thermo Fisher Scientific, Waltham, MA, USA). Statistical analyses were performed using Microsoft Excel 2013 (Microsoft Corporation, Redmond, WA, USA), SigmaPlot 12.3 (Systat Software Inc., San Jose, CA, USA), and IBM SPSS Statistics 23 (IBM Corporation, Armonk, NY, USA).

## 4. Conclusions

Considering the rising production, usage, and possible release of carbonaceous and BN NMs and the complexity related to their potential hazards, it is imperative that high-throughput screening approaches are developed to systematically assess the relative effects of NMs on various important biological receptors. NM testing in more complex systems also requires homogeneous and well-dispersed NM stocks to ensure reproducibility of the test results. Here, a protocol was optimized for preparing stable NM dispersions via ultrasonication using the environmentally relevant and biocompatible AA as a dispersant. The AA-aided dispersion protocol is universal for both carbonaceous and BN NMs, although the degree of stability varied across different materials with different physiochemical characteristics related to dimensions, morphologies, and sizes. The obtained AA-dispersed NM stock dispersions can be used for approaching homogeneous exposures of well-defined NM concentrations during microbial toxicity testing. The use of well-dispersed stocks improves the reproducibility of the toxicity results and facilitates data comparisons across studies. Overall, this work contributes to the methodology of NM toxicity research which can help improve the mechanistic understanding of NM dose-response and structure-activity relationships. 

## Figures and Tables

**Table 1:** Diameters of pristine and alginic acid (AA)-dispersed multiwall carbon nanotubes (CNT) CNT-1, CNT-2, and CNT-3, graphene nanoplatelets (GNP) GNP-1 and GNP-2, boron nitride nanotubes (BNNT), and hexagonal boron nitride flakes (hBN), and zeta (ζ) potentials of AA-dispersed nanomaterials in Nanopure water, Dryl’s medium, and half-strength 21C medium.

	Diameter (nm) ^1^		ζ Potential (mV) ^3^		
	Pristine ^2^	AA-Dispersed	Nanopure Water	Dryl’s	Half-Strength 21C
CNT-1	23.3 ± 5.5	21.4 ± 6.6	−53.1 ± 0.5	−34.8 ± 0.5	−35.8 ± 1.0
CNT-2	18.8 ± 4.1	13.8 ± 3.3	−44.2 ± 0.4	−32.9 ± 0.6	−31.1 ± 1.1
CNT-3	7.4 ± 1.9	8.3 ± 1.9	−36.4 ± 0.8	−27.9 ± 0.5	−32.4 ± 1.5
GNP-1	2415 ± 983	1847 ± 656	−52.5 ± 0.1	−40.6 ± 0.6	−39.7 ± 0.6
GNP-2	350 ± 320	1225 ± 797	−49.4 ± 0.2	−29.2 ± 0.3	−32.7 ± 0.3
BNNT	21.9 ± 14.7	15.3 ± 8.5	−56.9 ± 0.7	−35.4 ± 1.1	−33.5 ± 2.0
hBN	1796 ± 1098	306 ± 504	−65.3 ± 0.6	−40.0 ± 0.4	−36.7 ± 1.7

^1^ Measured from transmission electron microscopy (TEM) images. For CNTs and BNNTs, the diameters shown here are the outer diameters of the nanotubes. For GNPs and hBN, the diameters indicate one dimension of the two-dimensional sheets with thicknesses in the range of a few nm after exfoliation by sonication (data not shown). The thicknesses of the pristine GNPs were reported as <5 nm (GNP-1) and 8–12 nm (GNP-2), respectively (Table S1); ^2^ Diameters of pristine CNT-1, CNT-2, CNT-3, GNP-1, and GNP-2 have been published previously [21,34]; ^3^ Nanomaterial stocks (200 mg·L^−1^) dispersed with AA (400 mg·L^−1^) using 30 min of probe-sonication were diluted into either Nanopure water, Dryl’s medium, or half-strength 21C medium at a final concentration of 10 mg·L^−1^ for ζ potential measurement, with data shown as mean ± SE (*n* = 3).

## Supplementary Materials

The following are available online at http://www.mdpi.com/2079-4991/8/2/76/s1, Figure S1: Stock dispersions of alginic acid (AA)-dispersed multiwall carbon nanotubes (CNT) (a) CNT-1, (b) CNT-2, and (c) CNT-3, graphene nanoplatelets (GNP) (d) GNP-1 and (e) GNP-2, (f) boron nitride nanotubes (BNNT), and (g) hexagonal boron nitride flakes (hBN). Nanomaterial stocks (200 mg·L^−1^) were prepared by mixing with AA (400 mg·L^-1^) in Nanopure water followed by 30 min of probe-sonication (Scheme 1a, main manuscript), Figure S2: Linear correlation between dispersion optical density at 600 nm (OD_600_) and the nanomaterial (NM) concentration. NMs include multiwall carbon nanotubes (CNT) CNT-1, CNT-2, and CNT-3, graphene nanoplatelets (GNP) GNP-1 and GNP-2, boron nitride nanotube (BNNT), and hexagonal boron nitride flakes (hBN). NM stocks (200 mg·L^−1^) were prepared by mixing with alginic acid (AA, 400 mg·L^−1^) in Nanopure water followed by 30 min of probe-sonication (Scheme 1a, main manuscript). Then the stocks were diluted into Nanopure water to achieve a series of AA-dispersed NM dilutions containing 0, 10, 50, and 100 mg·L^−1^ of NMs. The diluted dispersions were measured for OD_600_ immediately following dilution. For each concentration, there were three replicates, but they appeared overlapped in the figure. The linear regression coefficient r was higher than 0.99 (all *p* < 0.001) for all seven NMs, Figure S3: Significant linear correlations between the stability of alginic acid (AA)-dispersed nanomaterials (NMs) in Nanopure water, indicated by the relative optical density at 600 nm (OD_600_) on Day 1 (blue triangles) and Day 7 (orange triangles) as compared to the OD_600_ at time 0 of the experiment, and either (a,c) the outer diameter or (d) diameter (Table 1) or (b) the specific surface area (Table S1) of the pristine NMs. (a,b) depict correlations established for multiwall carbon nanotubes (CNT-1, CNT-2, and CNT-3), (c) correlations for all one-dimensional NMs, including all three CNTs and boron nitride nanotubes (BNNT), and (d) correlations for all two-dimensional NMs, including two graphene nanoplatelets (GNP-1 and GNP-2) and hexagonal boron nitride flakes (hBN). The relative OD_600_ was used as a proxy for the percentage of AA-dispersed NMs remained dispersed (Figure 4a). For each NM, there were three replicates, but some of them appeared overlapped in the figure. The correlation results are shown in the figure as dashed regression lines with correlation coefficients (r) and significances (*p*). The dispersion protocol followed the steps for preparing AA-dispersed NM stock in Nanopure water by ultrasonication (Scheme 1a, main manuscript), Figure S4: The particle size histogram of pristine nanomaterials (NMs) and alginic acid (AA)-dispersed NMs measured from transmission electron microscopy (TEM) images. AA-dispersed NM stocks were prepared according to Scheme 1a, and then AA-dispersed NM powders were prepared from the stock dispersions as described in Materials and Methods (main manuscript). Pristine NM and AA-dispersed NM powders were then dispersed for TEM as described in Materials and Methods (main manuscript). The average diameters of NMs were derived from measuring over 100 particles of each NM within a representative area. A red solid line is a Gaussian fit. NMs include multiwall carbon nanotubes (CNT) CNT-1, CNT-2, and CNT-3, graphene nanoplatelets (GNP) GNP-1 and GNP-2, boron nitride nanotube (BNNT), and hexagonal boron nitride flakes (hBN), Figure S5: Stability of 100, 400, and 2000 mg·L^−1^ alginic acid (AA)-dispersed multiwall carbon nanotubes (CNT) CNT-1 in (a,b) Dryl’s medium and (c,d) half-strength 21C medium over one day, as measured by the time course of (a,c) the relative optical density at 600 nm (OD_600_, as a proxy for dispersed particle concentration) as compared to the OD_600_ at time 0 of the experiment, and (b,d) hydrodynamic diameter as measured by dynamic light scattering (DLS). Data bars are mean ± SE (*n* = 3). Note that some error bars (a,c) do not appear on the graph due to their small values, Table S1: Physicochemical characteristics of pristine multiwall carbon nanotubes (CNT) CNT-1, CNT-2, and CNT-3, graphene nanoplatelets (GNP) GNP-1 and GNP-2, boron nitride nanotubes (BNNT), and hexagonal boron nitride flakes (hBN), reported by the manufacturer, Table S2: Electrophoretic mobility (EPM) of alginic acid (AA)-dispersed multiwall carbon nanotubes (CNT) CNT-1, CNT-2, and CNT-3, graphene nanoplatelets (GNP) GNP-1 and GNP-2, boron nitride nanotube (BNNT), and hexagonal boron nitride flakes (hBN), Table S3: Properties of the D, G, and 2D bands of Raman spectra of pristine multiwall carbon nanotubes (CNT-1, CNT-2, and CNT-3), alginic acid (AA)-dispersed CNT (CNT-1-AA, CNT-2-AA, and CNT-3-AA), pristine graphene nanoplatelets (GNP-1 and GNP-2), and AA-dispersed GNP (GNP-1-AA and GNP-2-AA). I_D_/I_G_ and I_2D_/I_G_ represent the ratios of the D and G band intensities and of the 2D and G band intensities, Table S4: Assignments of Fourier transform infrared (FT-IR) spectral peaks of alginic acid (AA), pristine boron nitride nanotubes (BNNT), pristine hexagonal boron nitride flakes (hBN), AA-dispersed BNNT (BNNT-AA), and AA-dispersed hBN (hBN-AA).
