# Supplementary material for: Alginic Acid-Aided Dispersion of Carbon Nanotubes, Graphene, and Boron Nitride Nanomaterials for Microbial Toxicity Testing
Source: Nanomaterials (Basel). 2018 Jan 30;8(2):76. doi: 10.3390/nano8020076 (PMC5853708; doi:10.3390/nano8020076)
Supplement: Supplementary file 1 [file nanomaterials-08-00076-s001.pdf]

Supplementary Materials

Alginic Acid-Aided Dispersion of Carbon Nanotubes, Graphene, and Boron Nitride Nanomaterials for Microbial Toxicity Testing

Ying Wang [1], Monika Mortimer [1], Chong Hyun Chang [2] and Patricia A. Holden [1,*]

[1] Bren School of Environmental Science and Management, Earth Research Institute, University of California Center for Environmental Implications of Nanotechnology, University of California, Santa Barbara, CA 93106, United States; ywang@bren.ucsb.edu (Y.W.); mmortimer@bren.ucsb.edu (M.M.)
[2] University of California Center for Environmental Implications of Nanotechnology, California NanoSystems Institute, University of California, Los Angeles, CA 90095, United States; chonghyun@ucla.edu
* Correspondence: holden@bren.ucsb.edu; Tel.: +1-805-893-3195

Received: 01 December 2017; Accepted: 23 January 2018; Published: 30 January 2018

Table S1. Physicochemical characteristics of pristine multiwall carbon nanotubes (CNT) CNT-1, CNT-2, and CNT-3, graphene nanoplatelets (GNP) GNP-1 and GNP-2, boron nitride nanotubes (BNNT), and hexagonal boron nitride flakes (hBN), reported by the manufacturer [1].

Sample	Catalog #	Size (nm)	Specific Surface Area ($m^2 \cdot g^{-1}$)	Purity (wt.%)
CNT-1	Sku-030106	Diameter: 30–50, Length: 10000–20000	60	>95
CNT-2	Sku-030104	Diameter: 20–30, Length: 10000–30000	110	>95
CNT-3	Sku-030101	Diameter: <8, Length: 10000–30000	500	>95
GNP-1	GNP Grade 4	<4 layers, Thickness: <5	70	N/A
GNP-2	GNP Grade 3	Diameter: 2000, Thickness: 8-12	600–750	>97
BNNT	802824	Diameter: 5 ± 2	>100	>50
hBN	255475	Diameter: ~1000	N/A	98

[1] Information about three CNTs and two GNPs has been published previously [1,2]. N/A not available.

Table S2. Electrophoretic mobility (EPM) of alginic acid (AA)-dispersed multiwall carbon nanotubes (CNT) CNT-1, CNT-2, and CNT-3, graphene nanoplatelets (GNP) GNP-1 and GNP-2, boron nitride nanotube (BNNT), and hexagonal boron nitride flakes (hBN) [1].

	EPM ($\mu m \cdot cm \cdot V^{-1} \cdot s^{-1}$)		
	Nanopure water	Dryl's	Half-strength 21C
CNT-1	-3.77 ± 0.04	-2.47 ± 0.04	-2.54 ± 0.07
CNT-2	-3.14 ± 0.03	-2.33 ± 0.05	-2.20 ± 0.08
CNT-3	-2.58 ± 0.06	-1.98 ± 0.03	-2.30 ± 0.11
GNP-1	-3.72 ± 0.01	-2.88 ± 0.04	-2.82 ± 0.04
GNP-2	-3.50 ± 0.01	-2.07 ± 0.02	-2.32 ± 0.02
BNNT	-4.46 ± 0.06	-2.78 ± 0.08	-2.62 ± 0.15
hBN	-5.12 ± 0.05	-3.13 ± 0.03	-2.88 ± 0.13

[1] All nanomaterials (200 $mg \cdot L^{-1}$) were dispersed with AA (400 $mg \cdot L^{-1}$) using 30 min of probe-sonication and diluted into either Nanopure water, Dryl's medium, or half-strength 21C medium at a final concentration of 10 $mg \cdot L^{-1}$ (Scheme 1) for EPM measurement. All data are shown as mean ± SE (n = 3).

Table S3. Properties of the D, G, and 2D bands of Raman spectra of pristine multiwall carbon nanotubes (CNT-1, CNT-2, and CNT-3), alginic acid (AA)-dispersed CNT (CNT-1-AA, CNT-2-AA, and CNT-3-AA), pristine graphene nanoplatelets (GNP-1 and GNP-2), and AA-dispersed GNP (GNP-1-AA and GNP-2-AA). I_D/I_G and I_{2D}/I_G represent the ratios of the D and G band intensities and of the 2D and G band intensities [1].

Sample	Wavenumber (cm^{-1})			I_D/I_G	I_{2D}/I_G
	D	G	2D		
CNT-1	1328 ± 2	1576 ± 2	2644 ± 1	1.11 ± 0.07	0.78 ± 0.02
CNT-1-AA	1323 ± 1	1572 ± 1	2644 ± 1	1.02 ± 0.03	0.72 ± 0.01*
CNT-2	1332 ± 1	1586 ± 1	2655 ± 2	1.59 ± 0.02	0.56 ± 0.01
CNT-2-AA	1332 ± 2	1587 ± 2	2655 ± 3	1.79 ± 0.06*	0.42 ± 0.03*
CNT-3	1328 ± 0	1580 ± 1	2612 ± 2	0.13 ± 0.02	0.24 ± 0.01
CNT-3-AA	1329 ± 2	1588 ± 2*	2633 ± 5*	0.46 ± 0.12*	0.23 ± 0.02
GNP-1	1334 ± 1	1573 ± 2	2665 ± 4	0.23 ± 0.05	0.42 ± 0.02
GNP-1-AA	1335 ± 2	1582 ± 1**	2683 ± 1*	0.40 ± 0.03*	0.40 ± 0.00
GNP-2	1327 ± 2	1575 ± 2	2643 ± 1	0.83 ± 0.06	0.34 ± 0.01
GNP-2-AA	1327 ± 1	1571 ± 1	2650 ± 2*	0.65 ± 0.08	0.38 ± 0.02

[1] All data are shown as mean ± SE ($n = 3$). * $p < 0.05$ and ** $p < 0.01$, compared between the corresponding pristine nanomaterials (NMs) and the AA-dispersed NMs (for example, CNT-1 vs. CNT-1-AA). The dispersion protocol followed the steps for preparing AA-dispersed NM stock in Nanopure water by ultrasonication (Scheme 1a, main manuscript). NM powders for Raman analysis were prepared from the stock dispersions as described in Materials and Methods (main manuscript).

Table S4. Assignments of Fourier transform infrared (FT-IR) spectral peaks of alginic acid (AA), pristine boron nitride nanotubes (BNNT), pristine hexagonal boron nitride flakes (hBN), AA-dispersed BNNT (BNNT-AA), and AA-dispersed hBN (hBN-AA) [1].

Peak No.	Peak Assignment	Peak Wavenumber (cm^{-1})				
		AA	BNNT	BNNT-AA	hBN	hBN-AA
1	-OH, H-bonds	3367 ± 3	3214 ± 2	3216 ± 3	ND	3385 ± 2
2	C-H (CH_2)	2917 ± 1	ND	2920 ± 1	ND	2923 ± 1
3	C=O stretching vibrations (-COOH)	1718 ± 0	ND	1731 ± 1	ND	1733 ± 1
4	B-N (in plane)	ND	1354 ± 4	1339 ± 0	1326 ± 5	1343 ± 3
5	C-C-H and O-C-H deformation of pyranose rings	1235 ± 2	ND	ND	ND	ND
6	C-O-C and C-OH stretching of pyranose rings	1168 ± 0	ND	1117 ± 0	ND	1118 ± 1
7	C-O and C-C stretching vibrations of pyranose rings	1076 ± 0	ND	1081 ± 2	ND	1100 ± 0
8	C-O stretching vibrations	1029 ± 0	ND	1025 ± 0	ND	1028 ± 0
9	C-O stretching vibrations of uronic acid residue	941 ± 0	ND	941 ± 0	ND	941 ± 1
10	C-O stretching vibrations of uronic acid residue	927 ± 0	ND	922 ± 0	ND	922 ± 0
11	Guluronic acid block	876 ± 0	ND	ND	ND	ND
12	Mannuronic acid block	808 ± 1	ND	ND	ND	ND
13	B-N (out of plane)	ND	787 ± 5	767 ± 0	762 ± 1	759 ± 0

[1] All data are shown as mean ± SE ($n = 3$). ND = peak not detected. The dispersion protocol followed the steps for preparing AA-dispersed NM stock in Nanopure water by ultrasonication (Scheme 1a, main manuscript). NM powders for FT-IR analysis were prepared from the stock dispersions as described in Materials and Methods (main manuscript).

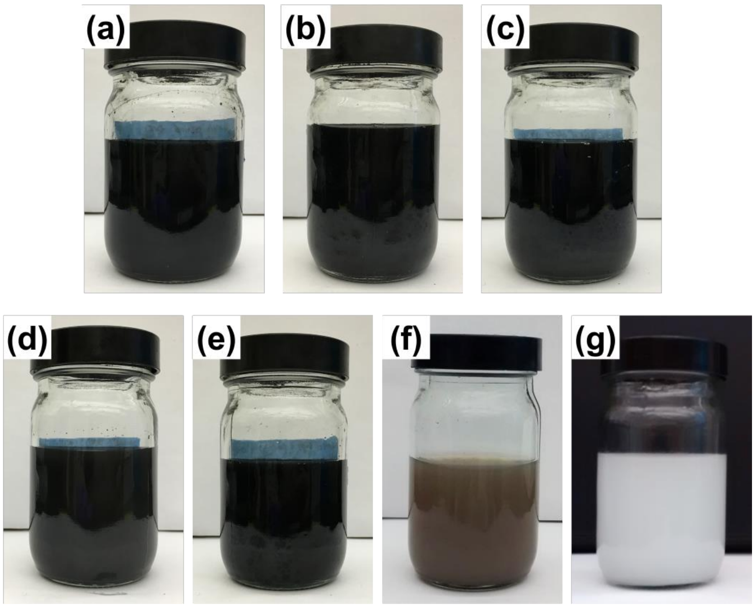

Figure S1. Stock dispersions of alginic acid (AA)-dispersed multiwall carbon nanotubes (CNT) (**a**) CNT-1, (**b**) CNT-2, and (**c**) CNT-3, graphene nanoplatelets (GNP) (**d**) GNP-1 and (**e**) GNP-2, (**f**) boron nitride nanotubes (BNNT), and (**g**) hexagonal boron nitride flakes (hBN). Nanomaterial stocks (200 mg·L^{-1}) were prepared by mixing with AA (400 mg·L^{-1}) in Nanopure water followed by 30 min of probe-sonication (Scheme 1a, main manuscript).

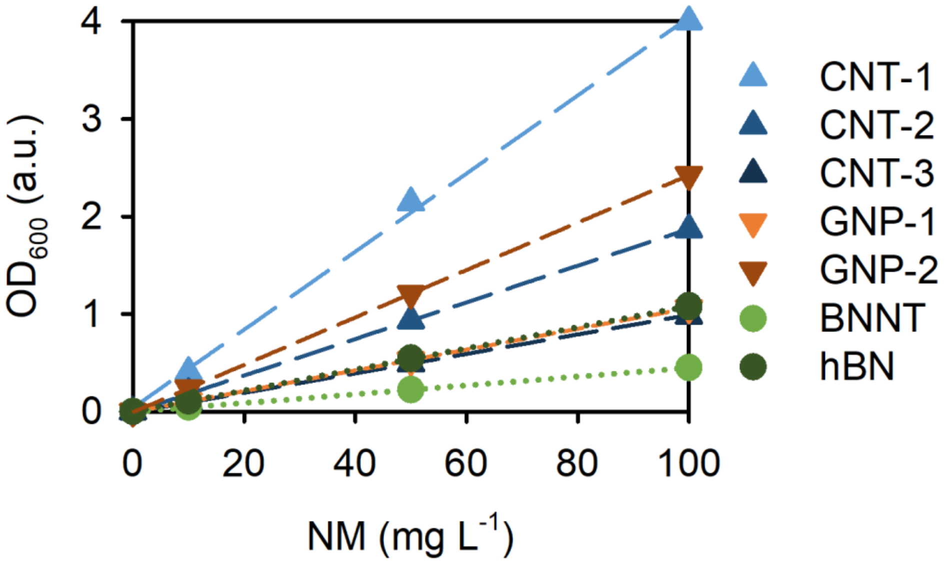

Figure S2. Linear correlation between dispersion optical density at 600 nm (OD_{600}) and the nanomaterial (NM) concentration. NMs include multiwall carbon nanotubes (CNT) CNT-1, CNT-2, and CNT-3, graphene nanoplatelets (GNP) GNP-1 and GNP-2, boron nitride nanotube (BNNT), and hexagonal boron nitride flakes (hBN). NM stocks (200 mg·L^{-1}) were prepared by mixing with alginic acid (AA, 400 mg·L^{-1}) in Nanopure water followed by 30 min of probe-sonication (Scheme 1a, main manuscript). Then the stocks were diluted into Nanopure water to achieve a series of AA-dispersed NM dilutions containing 0, 10, 50, and 100 mg·L^{-1} of NMs. The diluted dispersions were measured for OD_{600} immediately following dilution. For each concentration, there were three replicates, but they appeared overlapped in the figure. The linear regression coefficient r was higher than 0.99 (all p < 0.001) for all seven NMs.

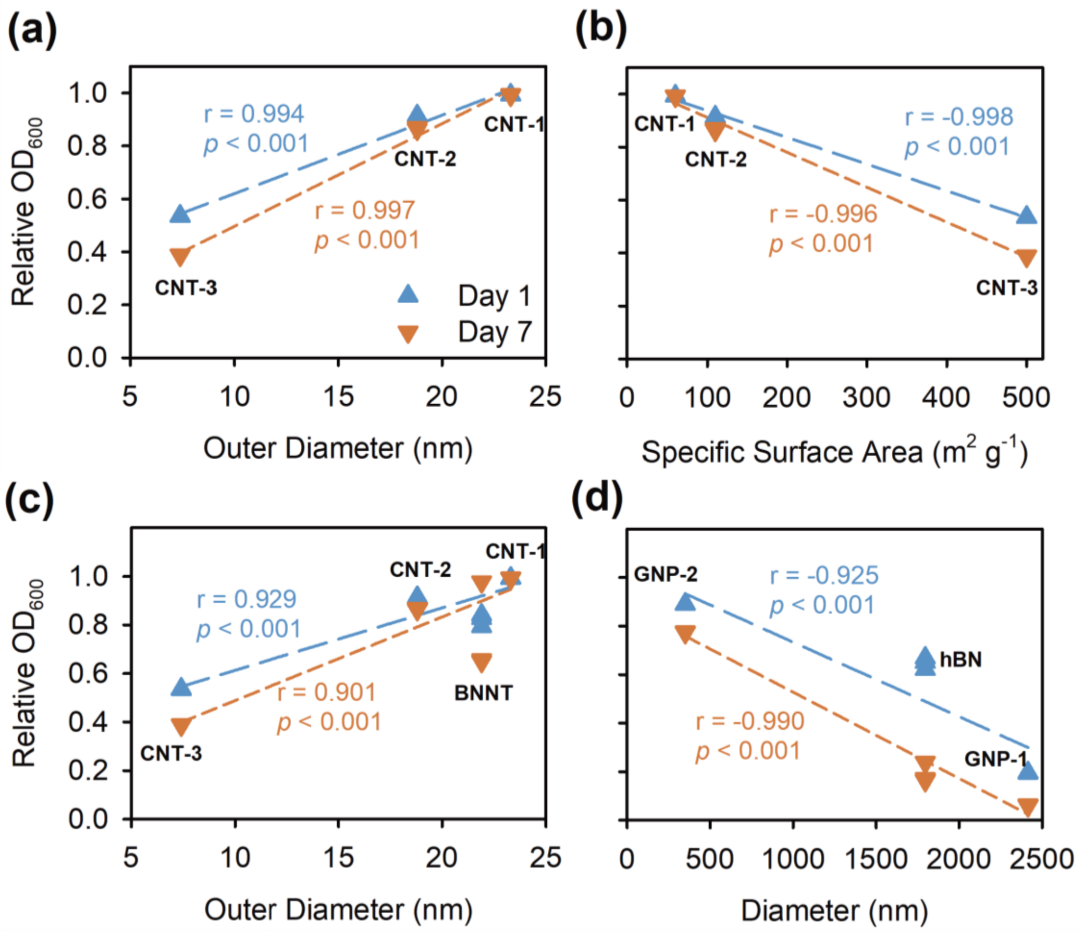

Figure S3. Significant linear correlations between the stability of alginic acid (AA)-dispersed nanomaterials (NMs) in Nanopure water, indicated by the relative optical density at 600 nm (OD_{600}) on Day 1 (blue triangles) and Day 7 (orange triangles) as compared to the OD_{600} at time 0 of the experiment, and either (**a**,**c**) the outer diameter or (**d**) diameter (Table 1) or (**b**) the specific surface area (Table S1) of the pristine NMs. (**a**) and (**b**) depict correlations established for multiwall carbon nanotubes (CNT-1, CNT-2, and CNT-3), (**c**) correlations for all one-dimensional NMs, including all three CNTs and boron nitride nanotubes (BNNT), and (**d**) correlations for all two-dimensional NMs, including two graphene nanoplatelets (GNP-1 and GNP-2) and hexagonal boron nitride flakes (hBN). The relative OD_{600} was used as a proxy for the percentage of AA-dispersed NMs remained dispersed (Figure 4a). For each NM, there were three replicates, but some of them appeared overlapped in the figure. The correlation results are shown in the figure as dashed regression lines with correlation coefficients (r) and significances (p). The dispersion protocol followed the steps for preparing AA-dispersed NM stock in Nanopure water by ultrasonication (Scheme 1a, main manuscript).

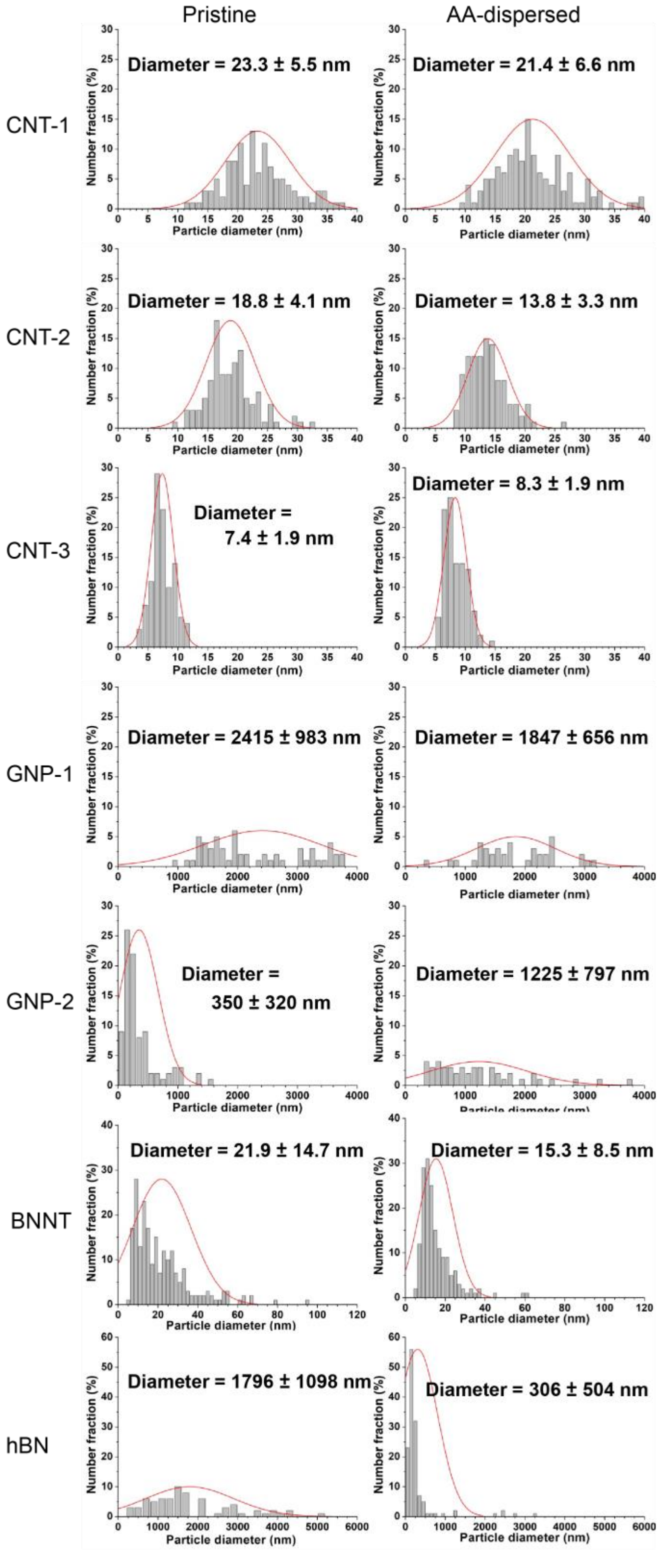

Figure S4. The particle size histogram of pristine nanomaterials (NMs) and alginic acid (AA)-dispersed NMs measured from transmission electron microscopy (TEM) images. AA-dispersed NM stocks were prepared according to Scheme 1a, and then AA-dispersed NM powders were prepared from the stock dispersions as described in Materials and Methods (main manuscript). Pristine NM and AA-dispersed NM powders were then dispersed for TEM as described in Materials and Methods (main manuscript). The average diameters of NMs were derived from measuring over 100 particles of each NM within a representative area. A red solid line is a Gaussian fit. NMs include multiwall carbon nanotubes (CNT) CNT-1, CNT-2, and CNT-3, graphene nanoplatelets (GNP) GNP-1 and GNP-2, boron nitride nanotube (BNNT), and hexagonal boron nitride flakes (hBN).

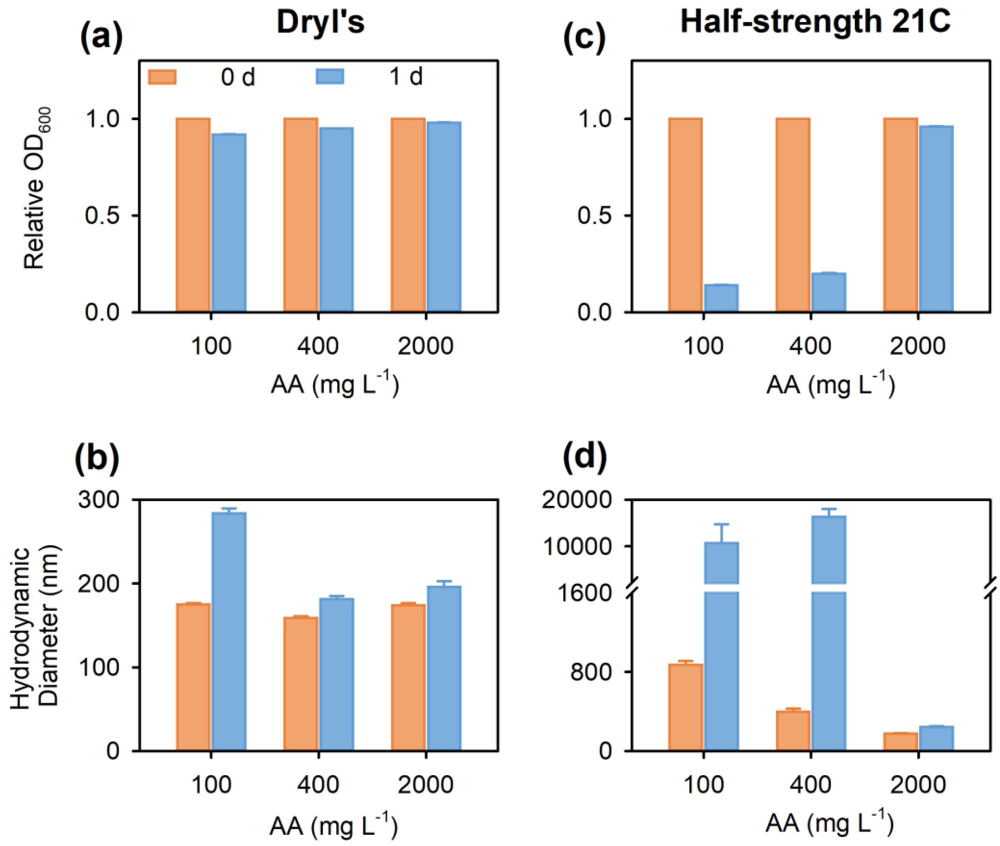

Figure S5. Stability of 100, 400, and 2000 mg·L^{-1} alginic acid (AA)-dispersed multiwall carbon nanotubes (CNT) CNT-1 in (**a**,**b**) Dryl's medium and (**c**,**d**) half-strength 21C medium over one day, as measured by the time course of (**a**,**c**) the relative optical density at 600 nm (OD_{600}, as a proxy for dispersed particle concentration) as compared to the OD_{600} at time 0 of the experiment, and (**b**,**d**) hydrodynamic diameter as measured by dynamic light scattering (DLS). Data bars are mean ± SE (n = 3). Note that some error bars (**a**,**c**) do not appear on the graph due to their small values.

References

1. Ge, Y.; Priester, J.H.; Mortimer, M.; Chang, C.H.; Ji, Z.; Schimel, J.P.; Holden, P.A. Long-term effects of multiwalled carbon nanotubes and graphene on microbial communities in dry soil. *Environ. Sci. Technol.* **2016**, *50*, 3965–3974, 10.1021/acs.est.5b05620.
2. Wang, Y.; Chang, C.H.; Ji, Z.; Bouchard, D.C.; Nisbet, R.M.; Schimel, J.P.; Gardea-Torresdey, J.L.; Holden, P.A. Agglomeration determines effects of carbonaceous nanomaterials on soybean nodulation, dinitrogen fixation potential, and growth in soil. *ACS Nano* **2017**, *11*, 5753–5765, 10.1021/acsnano.7b01337.

© 2018 by the authors. Submitted for possible open access publication under the terms and conditions of the Creative Commons Attribution (CC BY) license (http://creativecommons.org/licenses/by/4.0/).
